# Tumor-on-chip’s alliance with molecular pathology against metastatic disease

**DOI:** 10.1186/s12929-025-01209-8

**Published:** 2026-01-06

**Authors:** Emma Di Carlo

**Affiliations:** 1 Department of Medicine and Sciences of Aging, “G. d’Annunzio” University” of Chieti-Pescara, Via dei Vestini, 66100 Chieti, Italy; 2 Anatomic Pathology and Immuno-Oncology Unit, Center for Advanced Studies and Technology (CAST), “G. d’Annunzio” University of Chieti-Pescara, Via L. Polacchi 11, 66100 Chieti, Italy

**Keywords:** Tumor modeling, Metastasis, Organ-on-chip, Bioprinting, Personalized medicine, Molecular pathology

## Abstract

**Background:**

Cancer is the second leading cause of death worldwide. While significant progress has been made in early detection and treatment, metastasis remains the major cause of cancer-related morbidity and mortality. In the last decade the rate of long-term survivorship of metastatic cancer has continued to improve and overcoming resistance to therapy has now become a challenge. Developing strategies to prevent and treat metastatic disease is a priority for public health and requires a thorough understanding of the mechanisms driving progression of a specific patient's tumor and the rapid identification of targetable cancer drivers and drug resistance genes.

**Discussion:**

Custom bioprinted tumors, which recreate the interactions between tumors and surrounding tissues, can be integrated into organ-on-chip platforms, and leveraging molecular pathology and OMICS data, can provide highly realistic patient-specific models. These biomimetic tools enable the investigation of metastasis organotropism, the identification of therapeutic targets and the design of drug administration protocols to prevent metastasis and to overcome resistance. Benefits, limitations, and challenges to address for an efficient and routine application of this cutting-edge approach, together with the role of Artificial-Intelligence (AI) in managing the complex datasets generated by OMICS technologies will be highlighted in this review, as well as their real-life implications and evolutionary prospects.

**Conclusion:**

Applying patient-derived bioprinted tumors and organs for clinical purpose and developing standardized 4D and 5D bioprinting protocols would allow assessment of cancer response to treatments in a dynamic and faithfully reconstructed microenvironment. Integration of advanced molecular diagnostics and multi-OMICS data, with customized small-scale tumor models, assisted by AI-powered tools, requires a multidisciplinary framework. This integrated approach can upgrade clinical management of metastatic diseases, by accelerating the identification of actionable biomarkers and resistance mechanisms for timely therapy adjustments, thus enabling tailored treatment regimens based on individual tumor behavior.

## Background

Metastasis, the process by which cancer cells spread from the primary tumor to distant sites, represents a significant public health challenge worldwide [1]. As cancer diagnosis increases, due to aging populations and improved detection methods, the number of metastatic patients rises [2, 3], as well as the demand for long-term treatments and management of drug resistance.

**Fig. 1:**
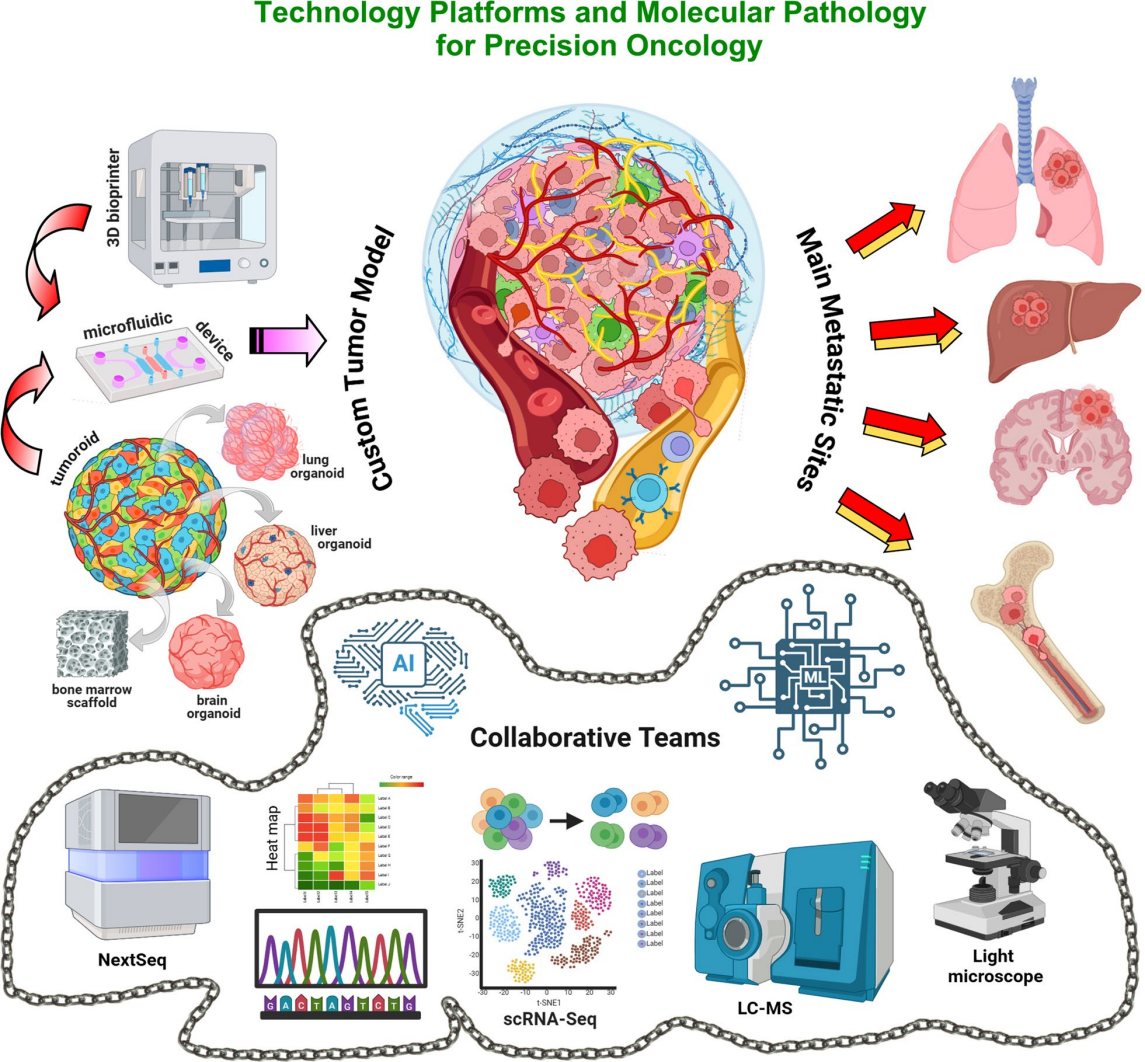
Synergy between technological platforms and artificial intelligence-assisted molecular biology and histopathology to advance precision oncology. Microfluidic device (on the left), containing either 3D patient-derived organoid or bioprinted tumor model (red arrows), connected by flow channels to miniatures of target organs of metastasis (represented by lung, liver, brain, and bone marrow organoids), can replicate architectural and functional features of the individual tumor (at the center) and the main metastatic sites (on the right). These cutting-edge platforms require AI- and ML-assisted histopathological and molecular investigations (light microscope, NextSeq, scRNA-seq, LC–MS) (at the bottom, included in a chain representing integration of advanced molecular diagnostics and multi-OMICS data) performed by a collaborative network of scientists and clinicians, to offer custom preclinical models for prognostic evaluation, drug testing and discovery, and to inform clinical decision making. This figure was created using BioRender (https://biorender.com/)

Innovative cancer care is needed, which requires investments, technologies and upgraded molecular diagnostics. The development of patient-derived tumor models [4] enabling modelling and simulation approaches, and reproducible pharmacokinetics (PK) and pharmacodynamics (PD), would facilitate prediction of clinical scenario and customized drug development. The integration of organ-on-chip (OOC, a multi-channel 3-dimension, 3D, microfluidic cell culture, that simulates an organ, or an organ system) and tumor-on-chip platforms (TOC, a specific application of the OOC technology designed to simulate cancer growth and invasiveness) [5, 6], bioprinting technology [7, 8], and molecular pathology, including AI-assisted OMICS sciences [9–11], may address this issue and would allow patient-tailored investigation and treatment of metastatic disease. The synergistic capabilities of these tools in mimicking human tumors, combined with functional genomics [12] and high-throughput drug screening, make this integrated approach a step forward in the development of precision oncology. The reasons for their synergy and their impact on research and on the progress of clinical practice are represented in Fig. 1 and summarized as follows.

1. *Accurate Recapitulation of the Tumor Microenvironment (TME) and High-Resolution Insights into Cancer Biology.* Metastasis is influenced by the TME [13]. TOC accommodate cancer, stromal and immune cells enabling their crosstalk, reproduce mechanical forces (pressure, stiffness) emulating the tumor’s real environment, and include microfluidic channels, which simulate blood flow and nutrient delivery [14, 15]. Bioprinting technology enables the construction of 3D tissue models with precise spatial organization [7] that closely mimic tumor architecture (the structural and spatial organization of the different components of a tumor) and microenvironments (complex ecosystems of the non-cancerous components interacting with tumor cells). Bio-fabricated 3D in vitro models on perfusable chips, create a miniature, living version of the tumour and of its surrounding environment, enabling the investigation of tumor progression steps [16]. Molecular techniques allow the identification of biomarkers for metastasis prediction and monitoring, the investigation of the TME's role in metastasis, and the assessment of the cellular and molecular components of the metastatic niches [17, 18], refining these platforms to better mimic tumour progression dynamics. Integration of genomic, transcriptomic, proteomic, and metabolomic data, through a multi-OMICS approach, offers insights into metastases-related molecular mechanisms and actionable targets [19]. By exploiting this data, bioprinting platforms can provide patient-tailored tumor bio-mimicking models, enabling personalized cancer research and drug testing [20, 21].
2. *Personalized Modeling of Metastatic Complexity*. Molecular pathology and OMICS provide insights into patient-specific molecular drivers of metastasis, including genetic mutations, protein markers, signaling pathways, gene expression profiles, and metabolic shifts [22, 23]. Bioprinting technology uses this information to build tumor constructs reflecting the unique biology of a patient’s primary tumor and metastasis [24, 25]. TOCs incorporate customized tumor models [26] and recreate the dynamic physical and chemical environments of tumors, including fluid flow, oxygen gradients, and mechanical forces, allowing for real-time testing of therapies targeting metastatic pathways [17]. This approach enables patient-tailored metastasis treatments and helps develop organ-specific targeting therapies.
3. *High-Throughput Drug Screening and Improved Monitoring of Therapy Response.* Metastatic cancer is often resistant to standard therapies and compels the discovery of new drugs and drug combinations. TOC devices enable testing of drugs under physiologically relevant conditions, such as shear stress and nutrient flow, which affect metastatic cells differently than primary tumor cells and allow, through imaging and sensors, for real-time monitoring of metastatic cell responses to therapy [27]. Bioprinting of vascularized tumor and organ models allows the investigation of how therapies affect tumor-vascular interactions and testing of multiple drugs across different metastatic contexts [28]. Molecular pathology identifies biomarkers of tumor resistance and sensitivity [29], ensuring that screening efforts focus on the most promising therapeutic targets, and offers post-therapy analysis of molecular changes in metastatic cells, to identify mechanisms of resistance and to inform the design of combination therapies. OMICS can power comprehensive datasets, which can be integrated with TOC data for systems biology, assisting predictive modeling of tumor behavior. This synergy identifying patient-specific molecular vulnerabilities and, predicting drug responses [30], accelerates the discovery of effective treatments and minimizes reliance on animal models.

Recent studies have provided evidence that TOC and OOC enable accurate predictions of drug efficacy and toxicity.

*Ewart et al.* developed a microfluidic human liver-on-chip for the prediction of drug induced liver injury. Testing 870 liver chips with 27 benchmark compounds, the authors report 87% sensitivity and 100% specificity, meeting qualification criteria from the Innovation and Quality (IQ) consortium. They also build an economic model showing that broad adoption could yield about 3 billion dollars/year in increased Research and Development (R&D) productivity by reducing unsafe drugs entering clinical trials [31]. *Jang et al.* built multispecies liver-chips (rat/dog/human), that recapitulate both human-specific and species-specific drug toxicities (e.g., compounds that are hepatotoxic in humans, but not in animals and *vice versa*). Their findings provided evidence that organ-chips can reveal human-relevant toxicity mechanisms that animal testing misses. [32]. *Chakrabarty et al.* developed a microfluidic cancer-on-chip that sustained viable tumour slices and accurately predicted treatment response in both breast and prostate cancer (PC) patient-derived xenograft (PDX) models exposed to cisplatin or apalutamide (compared with known *in vivo* responses). This is direct experimental validation that cancer-on-chip readouts reflect *in vivo* drug sensitivity [33].

A 2025 study, *by Pal et al.* describes a physiologically relevant, high-fidelity patient-matched esophageal adenocarcinoma (EAC) organ-chips platform containing organoids derived from a patient's own EAC tissue and patient-matched cancer-associated fibroblasts (CAFs). The EAC-chip reproduced the histology, and the genetics of the patient’s tumour, enabling the prediction of patient neoadjuvant chemotherapy responses (matched in-patient *Response Evaluation Criteria in Solid Tumors*, RECIST, outcomes) within a clinically useful timeframe (~ 12 days). Notably, direct concordance was found between chip response and the patient’s clinical objective responses [34].

Converging evidence prove that TOC systems, and integrated OOC networks, improve physiological relevance for both efficacy and safety testing and can bridge the gap between simple 2D assays, organoids, PDX models and clinical outcomes [35]. However, important limitations still hamper the real-life use of TOCs for prediction of treatment efficacy and toxicity, such as, *(a) Scale and prospective validation*. Several studies focused on TOC are proof-of-concept or limited-cohort. Large prospective clinical trials and multi-site qualification are still relatively few. A broader validation is needed before routine clinical use [26]. *(b) Throughput.* High-fidelity organ-chips are often lower throughput than plate-based organoids or 2D assays. Hybrid strategies (high-throughput organoids for screening and chips for follow-up) can be used to address this issue [34]. *(c) Standardization and regulatory acceptance.* Regulatory qualification for replacing animal studies is progressing (FDA modernization) [36], but broad regulatory acceptance requires standardized protocols and more cross-lab reproducibility data [31].

4. *Reduction in Animal Testing.* Animal models are often inadequate for studying the complexity of human metastatic disease due to species-specific differences in physiology, metabolism, and the complexities of disease progression, which limit the models' ability to accurately predict human outcomes. [37]. TOC and OOC, together with bioprinting platforms, can provide human-relevant models to improve clinical translatability of the research findings. Molecular pathology and OMICS ensure that these models are biologically accurate [29] for studying tumor behavior and testing drugs, thus reducing costs, and improving ethical standards. The latest advancements in these fields are outlined below.

## Organ-on-chip for modeling and targeting metastatic disease

OOCs are microfluidic devices consisting of microchannels operating on micro- to pico-liter volumes of fluid [6], that recreate the structure and function of human organs on a small, chip-like platform, to mimic the tissue interfaces, mechanical forces, and biochemical environment of specific organs (such as the lung, liver, heart, or kidney). Simulating blood vessels, microchannels generate a pulsatile flow, regulated by an external control unit that ensures the dynamic circulation of nutrients, drug, and waste transport in the cell culture chambers. Chambers, coated with extracellular matrix (ECM)-like components, house living cells derived from either primary tissue, cell lines or induced pluripotent stem cells, and are designed to ensure specific organ environments. Key components of an OOC device are described in Table 1.

**Table 1:** Key components of an Organ-on-a-Chip (OOC) device

Component	Function	Common materials	Examples/applications
*Microfluidic Channels*	Simulate fluid flow (e.g., blood, nutrients, or drugs)	PDMS^a^, glass, plastics (PMMA^b^, PC^c^, COP^d^, and COC^e^)	Networks for blood flow or drug delivery
*Cell Culture Chambers*	House cells/tissues to mimic organ functions	PDMS, hydrogels, ECM coatings	Alveolar-endothelial interface in lung-on-a-chip
*Porous Membranes*	Separate cell types, allow molecular exchange	Polycarbonate, polyester, PDMS	Gas exchange in lung chips, nutrient flow in gut models
*Pumps and Valves*	Regulate fluid movement within the device	External pumps, PDMS actuators	Simulate pulsatile or intermittent flow patterns, shear stress in vessels
*Stretchable Components*	Replicate mechanical forces (e.g., breathing, heartbeat)	Flexible PDMS, elastomers	Breathing simulation in lung-on-a-chip
*Sensors*	Monitor environmental parameters (pH, oxygen, metabolites, electrical activity)	Optical, electrochemical, mechanical	Real-time monitoring of tissue response or drug efficacy
*ECM Mimics*	Provide structural and biochemical support to cells	Collagen, fibronectin, agarose, alginate, PEG^f^, GelMA^g^, synthetic hydrogels	Mimic natural cell environments for tissue organization
*Inlet and Outlet Ports*	Introduce/remove fluids, drugs, or cells	PDMS, plastics	Precision control for introducing nutrients or drugs
*Scaffolds*	Offer 3D support for tissue growth and organization	Biodegradable polymers, hydrogels, or 3D-printed materials	Porous or lattice structures for organ-like tissue architecture and to promote cell migration and nutrient diffusion
*Integrated Electronics*	Facilitate electrical stimulation or activity recording	Microelectrodes, conductive polymers	Neural activity in brain-on-a-chip or cardiac simulations
*Housing and Encapsulation*	Protect internal components, maintain sterility	Transparent PDMS, glass, plastics	Ensure durability and optical access for imaging
*Imaging Windows*	Enable real-time visualization of cell behavior, chemical responses, other dynamic processes, and device function	Glass, transparent PDMS	Live cell imaging, such as fluorescence and confocal microscopy, or multi-photon microscopy. Label-free imaging, such as Raman microscopy, OCT^h^
*Drug Delivery Systems*	For precise, timed administration of test compounds	Inlet ports, microfluidic channels, reservoirs to maintain a steady supply for continuous delivery, valves and pumps	Mimic systemic drug exposure, or intermittent drug dosing, or site-specific treatments

^a^PDMS: polydimethylsiloxane

^b^PMMA: poly(methyl methacrylate)

^c^PC: polycarbonate

^d^COP: cyclic olefin polymers

^e^COC: cyclic olefin copolymers

^f^PEG: polyethylene glycol

^g^GelMA: gelatin methacrylate

^h^OCT: Optical Coherence Tomography

OOC technology can now be organically incorporated into the drug development pipeline, from discovery to preclinical screening, prior to approval for clinical trials by the U.S. Food and Drug Administration [38]. OOCs represent an advanced alternative to spheroids and traditional 2D cell cultures or animal models due to their potential to faithfully replicate at a small-scale human-specific cellular behaviors [4]. When reproducing 3D tumor microenvironment, including cancer, stromal, vascular, and immune components they are also known as TOCs that offer physiologically relevant models to study tumor growth, invasion, angiogenesis, and drug response. TOCs specifically engineered to model metastatic spread and colonization can incorporate, in addition to the primary tumor components, tissue-specific microenvironments (e.g., bone, liver, lung, or brain niches). These platforms require interconnected multi-tissue chips, facilitating cell migration and multi-organ crosstalk to study how circulating tumor cells adhere, extravasate, and grow at distant organ sites.

Metastasis is a hallmark of malignancy, accounting for over 90% of cancer-related deaths [39]. Its dynamics entail a multistep process [40] that includes, *local invasion* by which cancer cells break through the surrounding tissue; *intravasation* by which cells enter blood or lymphatic vessels; *survival and circulation* of spreading cells; *extravasation*, and *organ-specific colonization* of metastatic cells. These dynamics are shaped by cancer cell intrinsic factors, TME, immune system, physical and mechanical conditions, premetastatic niches, and extrinsic factors, such as treatments, age, and comorbidities. OOC provides a unique platform for an in-depth study of these tightly regulated pathways due to the features outlined below.

- *Modeling the TME*, by mimicking the 3D architecture and heterogeneity of tumors, which allows to investigate cancer cell crosstalk with surrounding fibroblasts, immune and endothelial cells (ECs). The stiffness and content in growth factors, metalloproteinases and immune mediators of ECM can be modulated [41] and the role of mechanobiology (the impact of mechanical cues, like stiffness, tension, and shear stress, on cell behavior, tissue organization and function) [42, 43] in metastasis can be explored in real-time [44]. The molecular mechanisms by which interstitial fluid pressure [45] or CAFs [46] regulate epithelial-mesenchymal transition (EMT) of breast cancer (BC) cells, promoting their migration and invasion have been investigated by using BC-on-chips. The impact of tumor stroma on drug penetration and efficacy can also be evaluated [47]. The effect of ovarian cancer-stromal fibroblasts cross-talk on drug resistance was investigated by using a 3D microvascularized multi-niche TOC. By using this five-chamber platform researchers demonstrated an increased drug resistance to carboplatin/paclitaxel in the presence of CAFs, when compared to normal fibroblasts, and the reversal of CAF-mediated drug resistance by ECM targeted therapy [48].
- *Enabling the study of invasion and intravasation*, since movements of cancer cells embedded into ECM-like gels, endowed with tunable mechanical properties, can be visualized by real-time imaging of cell behavior, and quantified in the chip [35]. Microfluidic chips integrate endothelial-lined microchannels to study cancer cell-vessel wall interactions and to investigate how inflammation, matrix degradation, and mechanical forces affect cancer cells’ ability to breach tissue barriers [49]. Micro-physiological systems (MPS), that combine microfluidics, 3D tissue culture, and organ-specific cell types, have been incorporated into the OOC platforms namely Invasion/Chemotaxis “IC-Chip” and extravasation “EX-Chip”, that provide assays mirroring in vivo tropism patterns. These chips quantify tissue-specific invasion and extravasation under defined flow/ECM and stromal conditions, providing evidence of the microenvironment-dependent mechanisms that regulate cancer cell trafficking, metastatic niche formation and organ-specific colonization [50–52].
- *Simulation of blood-flow dynamics and shear stress* to investigate the mechanisms by which circulating tumour cells (CTCs) survive, evade immune detection, interact with platelets or immune cells, and colonize distant organs. OOC can incorporate vessel network-like structures, enabling realistic modeling of cancer cell spreading to distant organs, immune cell trafficking and drug transport. Interaction of fluorescence labeled CTCs, which can be tracked with microscopy, with organ-specific endothelial barriers can be investigated to understand the different vulnerability of the organs to cancer cell colonization and metastases [53, 54].
- *Modeling metastatic microenvironments* to investigate cancer cell extravasation and colonization of specific organs. Organotropism of metastasis (the propensity of cancer cells derived from a primary tumor to colonize specific distant organs), results from complex interactions between cancer cell properties and target organ microenvironments [55]. Lung-on-a-Chip, that mimics the alveolar-capillary interface, is useful for studying metastases from different cancer types [56]; Liver-on-a-Chip, containing hepatocytes, sinusoidal ECs, and Kupffer cells, is useful for investigating metastasis from gastrointestinal and breast tumors [57]; Brain-on-a-Chip models, incorporating neurons, astrocytes, microglia, and blood-brain-barrier ECs, are useful for studying melanoma, lung and BC metastasis [58]; Bone-Marrow-on-a-Chip, incorporating a ceramic scaffold containing bone matrix proteins, hematopoietic cells and osteoblasts, is useful for investigating PC propensity for bone metastasis [59]; Recently, *Chen and co-workers* engineered a 3D bone-like microenvironment by integrating calcium phosphate scaffolds mimicking trabecular bone, decellularized ECM showing osteo-inductive properties, mesenchymal stem cells (MSCs), and osteoblasts. This biomimetic niche induced a proliferation-inhibited state in PC cells, closely mirroring the transcriptomic signatures identified from patient-derived single-cell (sc) RNA-seq datasets. In this niche, tumour cells displayed enzalutamide resistance, accompanied by metabolic reprogramming and activation of pro-survival signalling pathways for therapeutic evasion. This platform provides a clinically relevant tool for modelling PC bone metastasis and designing therapies targeting resistant tumours [60].

Contemporary platforms now incorporate 3D extracellular matrices, perfusable vascular and lymphatic networks, multiple interconnected organ compartments, and even patient-derived tumours tissues [61]. These advances allow OOC systems to capture key biological processes underlying metastasis with unprecedented control and resolution, as outlined below.

## Next-generation tumor-on-chip platform to recreate real-world metastasis

**Fig. 2:**
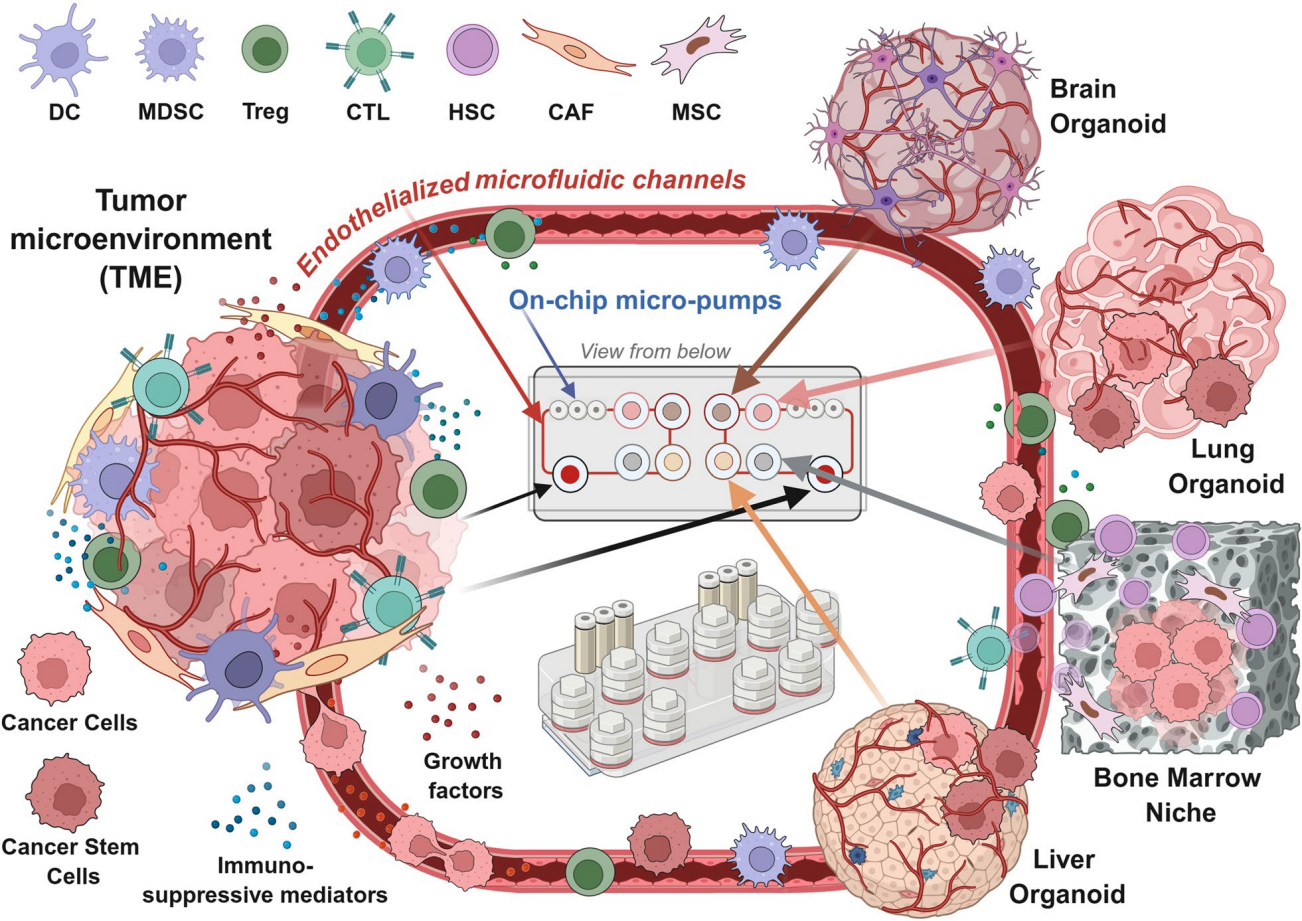
Patient-derived multi-organ-on-a-chip with autologous immune cells. Microfluidic bioreactor, multi-OOC (a), which houses in one compartment (*black arrows*) the 3D tumour spheroid, containing cancer and immune cells, CAFs, and microvessels (recapitulating the TME) connected, via microfluidic endothelialized channels (*red arrow)* to multiple miniaturized, engineered tissue compartments, each representing a different human organ. The Chip incorporates immune cells (DC: dendritic cell; MDSC: myeloid-derived suppressor cell; Treg: T regulatory cell; CTL: cytotoxic T lymphocyte) derived from the same individual into the tumour-organs shared circulation. The main organs where metastases occur are represented by—brain spheroid (*brown arrow*) consisting of co-cultured astrocytes and neurons—lung spheroid (*pink arrow*) made up of co-cultured pneumocytes and endothelial cells—bone scaffold embedded with bone marrow (BM)-derived mesenchymal stem cells (MSCs) and hematopoietic stem cells (HSCs), to mimic the BM niche, and liver spheroid formed by hepatocytes, Kupffer and stellate cells. Micropumps, generating a pulsatile flow (adjusted by a control unit), ensure the dynamic circulation of the culture medium between the compartments. This figure was created using BioRender (https://biorender.com/)

Recent evolutions in OOC design have significantly enhanced the ability to model the metastatic cascade, enabling real-time observation of tumor invasion, intravasation, circulation, and organ-specific colonization, providing more physiologically relevant tools for evaluating anti-metastatic therapies. Newer generation platforms, now include:

- *Multi-Organ-on-Chips*, designed to mimic organ-specific microenvironments and to model all the metastatic steps, enabling exploration of the role of the premetastatic niche and organ-specific microenvironments in cancer progression (Fig. 2) [62, 63]. Linked modules (tumours, circulation, and distant organ compartments, such as lung, liver, bone) allow the transit of tumours cells and systemic factors between compartments and enable modelling of organotropism and drug effects across organs. An organotropism model for circulating BC cells, based on a leaf-vein-based multi-organ microfluidic chip, has proven useful to explore their organ-specific adhesion propensity in common metastatic sites, such as liver, bone, and lung [64]. Modeling metastasis in multiple organs and their crosstalk with the primary tumor is fundamental for high-throughput drug screening [5, 44] enabling testing of a wide array of compounds simultaneously and assessing drug resistance mechanisms [65, 66]. *Xu *et al. used a multiorgan-on-a-chip platform to investigate lung cancer metastasis to three target organs [67]. The microfluidic chip was composed of an upstream compartment housing (A549) lung cancer cell (together with bronchial epithelial cells, macrophages, fibroblasts, ECs), separated via (biocompatible, transparent and gas permeable) polydimethylsiloxane (PDMS) microporous membranes, from downstream brain, bone, and liver compartments. The model allowed molecular investigations on migrating lung cancer cells and cell populations in the downstream compartments. Metastatic lung cancer cells reversed EMT markers (N-cadherin, Snail1, and Snail2), increased E-cadherin and, in brain-like microenvironment, upregulated CXCR4 expression. Bone metastasis induced osteolytic RANKL that activates cancer cell RANK, promoting tumor spread, while liver metastasis triggered hepatocyte AFP overexpression, indicating liver injury. In vivo imaging of tumor growth and progression in nude mice validated the metastatic behavior observed in the multi-OOC system, suggesting this platform is a valid tool to mimic cancer metastasis microenvironments and to investigate cell–cell interactions during metastasis.
- *Immune System-on-Chip,* designed to incorporate *(a) immune components*, such as T cells, NK cells, and macrophages, which can regulate cancer cell spreading [68], *(b) tissue-mimicking microenvironments*, and *(c) dynamic fluid flow to model immune responses*, such as inflammation, cell trafficking, antigen presentation, and cytokine signaling with high physiological relevance. This platform allows the study of immune editing (the process by which the immune system shapes tumour cells, selecting for variants that evade immune detection), immune-mediated remodeling of niches, and testing of how immunotherapies influence metastatic behavior (Fig. 2). Models of the liver and lungs (the most frequent sites of metastasis) have been integrated with immune cells [69, 70] to investigate their interaction with CTCs and micro-metastases. A patient-derived glioblastoma (GBM)-on-a-Chip model was developed using patient-derived glioblastoma cells and autologous regulatory T cells and myeloid-derived suppressor cells, to recapitulate the immunosuppressive GBM microenvironment. This microfluidic platform enabled real-time monitoring of tumor–immune interactions and dissected mechanisms of PD-1 checkpoint resistance. Using the chip, researchers identified strategies to enhance T-cell infiltration and cytotoxic activity, demonstrating its potential as a preclinical tool for personalized testing and optimization of PD-1–based immunotherapies in glioblastoma [71]. To evaluate tumor-immune dynamics, Campisi et coworkers developed a 3D microfluidic platform for ex vivo culture of murine- (CT26 colon carcinoma, B16 melanoma, Lewis lung carcinoma, GL261 glioblastoma) and patient- (ovarian cancer, non-small cell lung cancer) derived organotypic tumor spheroids, preserving tumor architecture and infiltrating immune cells. The system enables real-time analysis of immune checkpoint interactions, including PD-1/PD-L1 signaling, and allows testing of immunotherapies. It provides a physiologically relevant, high-throughput model to optimize checkpoint blockade strategies in a personalized manner [72].
- *Patient-derived Organoid-on-Chip* is being developed for testing patient-tailored tumor therapies. These platforms containing 3D tumor organoids [73] developed from cancer stem cells (CSCs), or tumoroids developed from cancer cells isolated from patient biopsies or resected tumors, can incorporate autologous immune and stromal cells. Organoids generated from (adult or pluripotent) stem cells (SCs) mimic organ sites of metastasis, and replicate an individual’s unique metastasis microenvironment, useful to predict the most effective treatment [74]. The transition from 2 to 3D cancer cell culture represents a paradigm shift in oncology research. While 2D systems remain useful for high-throughput screening (HTS) and basic mechanistic studies, 3D models offer superior physiological relevance, predictive accuracy, and translational value, as detailed in Table 2. 3D cancer cell cultures more accurately replicate the physiological conditions of in vivo tumors than traditional 2D monolayers. In 3D systems, cells grow in all directions, allowing realistic cell–cell and cell–ECM interactions that influence proliferation, differentiation, and gene expression. The spatial organization produces gradients of oxygen, nutrients, and signaling molecules that mimic tumor heterogeneity and microenvironmental stress. Consequently, 3D cultures exhibit drug diffusion barriers and resistance patterns closer to clinical behavior. They also support co-cultures with stromal or immune cells, enabling studies of tumor–microenvironment crosstalk. Overall, 3D culture systems, especially patient-derived organoids (PDOs), retain the genomic heterogeneity and drug-response profiles of the original tumor tissue, improving the accuracy of tumor modeling and the predictive power of clinical drug screening. This makes them invaluable for personalized medicine and biomarker discovery. In contrast, 2D cell cultures often lose key mutations and adaptive traits after repeated passaging [75]. Unlike traditional cell cultures, PDOs and patient-derived tumor organoids (PDTOs) retain the heterogeneity and complexity of peripheral organ and primary tumor microenvironments, respectively. Table 3 reports concrete examples of PDTO-on-chip platforms developed for tailoring tumour therapies [26, 76].
- *Chip integration with advanced readouts and computational intelligence* has become crucial in advancing OOC technology [77, 78]. Coupling chips to live fluorescence/confocal microscopy, embedded physical sensors (TEER, oxygen, pH) [79, 80], microdialysis, and downstream single-cell and bulk multi-omics (scRNA-seq, proteomics, metabolomics, mass spectrometry), provides mechanistic, spatiotemporal, and molecular data on metastatic cells (e.g., signalling states during extravasation), enables real-time monitoring of metastatic events, and links phenotype to gene/protein signature. Combining OOC experimental data with computational models (agent-based, fluid dynamics, machine learning, ML) [81, 82] to predict dissemination trajectories, optimize chip geometry, or interpret high-dimensional readouts, improves extrapolation of patient outcomes, and analysis of complex datasets from multi-organ chips [83]. Table 4 compares the characteristics of different OOC platforms for metastasis research.

**Table 2:** Characteristics and advantages of 3D *versus* 2D cell cultures

Parameter	2D cell culture	3D cell culture	Advantages of 3D
Architecture	Flat monolayer Limited spatial growth	Spheroid or scaffold Full 3D architecture	Mimics in vivo tumor morphology
Cell–Cell & Cell–ECM interaction	Minimal Mainly lateral contacts	Extensive, multidirectional interactions	Recreates tumor microenvironment
Gene expression	Altered, often non-physiological	Closely resembles in vivo gene expression profiles	Higher biological fidelity
Drug diffusion & response	Uniform drug exposure Overestimates efficacy	Gradient-dependent penetration	Predicts clinical resistance mechanisms
Microenvironment simulation	Absent or simplified	Includes hypoxia, pH, and nutrient gradients	Models tumor heterogeneity
Mechanical cues	Absent (rigid substrate)	Dynamic Tissue-like stiffness	Realistic mechanotransduction
Co-culture capability	Limited	Supports stromal, immune, and endothelial co-cultures	Captures tumor-stroma/immune crosstalk
Phenotypic stability	Rapid de-differentiation	Maintains differentiation and heterogeneity	Better mirroring of tumor behavior
Clinical relevance	Low translational predictability	High relevance. compatible with patient-derived organoids	Useful for personalized therapy
Applications	Basic mechanistic studies	Drug screening, tumor modeling, personalized medicine	Enhanced physiological and translational outcomes

**Table 3:** Patient-derived tumor organoid (PDTO)-on-chip platforms with therapeutic focus, validation status, and key findings

Tumor type	Platform and microenvironment features	Therapeutic focus/Assays performed	Validation status (clinical correlation)	Key findings	References
Hepatocellular carcinoma (HCC)	Multi-layer microfluidic high-throughput chip with microwell arrays; PDTOs co-cultured with MSCs, CAFs and autologous PBMCs to recreate TME and immune components	Drug screening including anti-PD-L1 immunotherapy and chemotherapies; immune-response assays	Partial clinical correlation shown: platform better correlated with patient immunotherapy outcomes than standard PDTO culture	Adding MSCs/immune cells improved PDTO establishment and better predicted immunotherapy responses compared with PDTOs alone	*Adv Sci (Weinh).* 2023 Sep;10(27):e2302640. https://doi.org/10.1002/advs.202302640
Colorectal cancer (CRC). Patient-derived CRC organoids	Two-channel microfluidic organ-on-chip generating fluid flow and rhythmic mechanical strain (to mimic peristalsis); epithelial + endothelial compartments; real-time permeability/invasion readouts	Invasion assays, mechano-biology studies, drug testing under physiologic mechanical forces	Preclinical; transcriptomic match to patient tumours reported; not yet shown as prospective clinical decision tool	Chip reproduced patient tumour transcriptional profiles better than PDTO alone; KRAS-mutant tumours displayed mechano-sensitive invasion and metabolic shifts (eg GABA usage)	*Trends Biotechnol. 2023 Mar;41(3):278–280. *https://doi.org/10.1016/j.tibtech.2023.01.004 *bioRxiv.* 2023 Sep 17:2023.09.14.557797. https://doi.org/10.1101/2023.09.14.557797
Multiple solid tumours (PDTO from various cancers)	Vascularized organoid-on-chip with tumor-specific hierarchical microvasculature (endothelial networks) enabling angiogenesis and intravasation modeling	Anti-angiogenic and anti-metastasis drug testing; assays of tumour–vascular interactions and migration	Preclinical with correlation of chip angiogenic/migratory behaviour to patients' metastatic outcomes	Highly metastatic PDTOs induced angiogenesis and migrated toward vessels via Notch signalling; platform useful for anti-vascular drug evaluation	*Nat Commun. 2024 Feb 16;15(1):1452. *https://doi.org/10.1038/s41467-024-45710-4 *Small.* 2024 Jul;20(27):e2308525. https://doi.org/10.1002/smll.202308525 *Adv Mater.* 2025 Feb;37(6):e2412815. https://doi.org/10.1002/adma.202412815
Pancreatic ductal adenocarcinoma (PDAC)	Tumour-chip incorporating patient-derived pancreatic organoids with pancreatic stellate cells (stromal) and macrophages; recapitulates PDAC TME and desmoplastic stroma	Gemcitabine and combination chemotherapy testing; stromal-targeting interventions; readouts of invasion, viability	Preclinical; model recapitulated key TME features and drug responses consistent with PDAC biology; not yet standard clinical diagnostic	Inclusion of stroma and immune components improved physiological relevance; platform showed patient-specific drug sensitivities	*Microsyst Nanoeng.* 2022 Mar 31;8:36. https://doi.org/10.1038/s41378-022-00370-6
Glioblastoma (GBM). PDTO and primary GBM cells used in chips	GBM-on-chip implementations integrate 3D tumour constructs with microfluidic gradients, ECM, and immune/vascular components; mimic invasion and blood–brain barrier interactions	Drug penetration, invasion, radiotherapy & chemo sensitivity assays; immunotherapy modelling in microenvironment	Mostly preclinical; several studies report improved recapitulation of invasion and therapy resistance vs 2D models; limited prospective clinical validation	GBM chips allow modelling of heterogeneity, invasion, and therapy resistance; useful for mechanistic study and testing patient-matched regimens in research setting	*Front Oncol.* 2023 Jul 12;13:1183059. https://doi.org/10.3389/fonc.2023.1183059
Breast cancer (PDTO integrated into chip)	Modular tumor-chip with multiple compartments enabling co-culture of breast cancer PDTOs and immune/stromal cells; used to test CAR-T cell activity and drug responses	Immune-cell (CAR-T) efficacy testing, chemotherapy/targeted agent screens, immunotherapy modelling	Preclinical translational work; demonstrated patient-specific CAR-T activity ex-vivo but not yet used prospectively to select patient therapies	Platform supported testing of patient tumor sensitivity to CAR-T and drugs; promising for personalized immunotherapy screening	*Cell Stem Cell.* 2024 Jul 5;31(7):989–1002.e9. https://doi.org/10.1016/j.stem.2024.04.018
Breast cancer (PDTO formation and manipulation)	Microfluidic device enabling high-fidelity formation of breast cancer organoids with addressable release for downstream assays; amenable to integration with multi-organ chips	High-throughput organoid formation, morphology-dependent growth studies, downstream drug screening workflows	Engineering/preclinical tool that improves throughput and handling of patient organoids to accelerate downstream testing. Supports PDTO-on-chip pipelines	Improves generation, shape control and harvest of PDTOs for subsequent drug testing; useful as upstream module for PDTO-on-chip workflows	*Adv Mater.* 2024 Nov;36(44):e2410547. https://doi.org/10.1002/adma.202410547
Lung cancer (patient-derived lung cancer organoids, sometimes combined with chips)	Studies describe generation of large lung PDTO cohorts and integration strategies with microfluidic platforms/functional assays. Immune-inclusive PDTO chips. Lung-on-chip adaptations	High-throughput drug screening, targeted therapy sensitivity, immune-co-culture assays (PD-1/PD-L1), ex-vivo prediction of patient response	Several real-world studies show PDTO drug responses correlate with patient outcomes (lung PDTO panels); chip integration mostly preclinical but rapidly maturing	Lung PDTOs have been used to predict responses in clinical cohorts; organoid-on-chip adaptations aim to add flow/mechanics and immune/stroma to further improve predictive value	*Front Bioeng Biotechnol.* 2023 May 26;11:1,205,157. https://doi.org/10.3389/fbioe.2023.1205157 *Cell Oncol (Dordr).* 2023 Jun;46(3):503–519. https://doi.org/10.1007/s13402-023-00771-3 *Adv Sci (Weinh).* 2024 Aug;11(31):e2400185. https://doi.org/10.1002/advs.202400185 *Front Cell Dev Biol.* 2025 Apr 15;13:1554268. https://doi.org/10.3389/fcell.2025.1554268

**Table 4:** Comparison between OOC platforms for metastasis research

Platform	Metastatic step(s) modeled	Complexity	Throughput	Typical readouts	Key citations
Tumor-on-chip (single-organ microfluidic)	Local invasion, matrix remodeling, short-range migration, drug response in TME	Medium (device + 3D ECM + stromal cells)	Medium	Live-cell imaging of invasion, transmigration assays, matrix degradation (zymography), secreted factors	*Proc Natl Acad Sci U S A.* 2015 Jan 6;112(1):214–9. https://doi.org/10.1073/pnas.1417115112
Vascularized tumor-on-chip (perfusable endothelium)	Intravasation, adhesion to endothelium, intravascular survival, extravasation under shear	High (endothelialization + flow control)	Low–Medium	Time-lapse imaging of transendothelial migration, shear-dependent adhesion assays, TEER/permeability, immunostaining	*Anal Chem.* 2022 Sep 6;94(35):12159–12166. https://doi.org/10.1021/acs.analchem.2c02556
Multi-organ/metastasis-on-a-chip (linked modules)	Full dissemination cascade, organotropism, pre-metastatic niche formation	Very high (multiple organ compartments, interconnecting channels)	Low	Tracking of circulating tumor cells (CTCs), organ-specific colonization, endpoint histology and organ-specific biomarkers, multi-omics of seeded cells	*Front Oncol.* 2025 May 29;15:1,602,225. https://doi.org/10.3389/fonc.2025.1602225
*Patient-derived Organoid-on-Chip*	Patient-relevant invasion, heterogeneity in intravasation/colonization, drug response predictive assays	High (PDO generation + chip integration)	Low	Functional assays (growth, invasion), single-cell RNA-seq after dissemination, drug sensitivity (viability), morphology	*iScience.* 2023 Sep 29;26(10):108,094. https://doi.org/10.1016/j.isci.2023.108094
Immune/immune-tumor-on-chip	Immune surveillance of CTCs, immune-mediated clearance/support of extravasation, immunotherapy testing	High (immune cell sourcing + dynamic co-culture)	Low	Immune cell trafficking & killing assays, cytokine profiling, flow cytometry, longitudinal imaging	*Proc Natl Acad Sci U S A.* 2018 Jul 3;115(27):7022–7027. https://doi.org/10.1073/pnas.1715932115
Bone/osteolytic niche chips	Bone colonization, osteolysis, tumor–osteoblast/osteoclast interactions (breast, prostate metastasis)	High (mineralized matrix + bone cells)	Low	TRAP activity, mineralization assays, osteoclast/osteoblast markers, imaging of colonization	*Proc Natl Acad Sci U S A.* 2018 Feb 6;115(6):1256–1261. https://doi.org/10.1073/pnas.1714282115
3D bioprinting + OOC hybrids	Controlled architecture for invasion/angiogenesis, perivascular niche formation, spatially patterned ECM effects	Medium–High (printing + microfluidics)	Medium	Structural fidelity (microCT/IF), invasion along printed ECM, vascular network formation, reproducibility metrics	*Nat Commun.* 2023 Nov 24;14(1):7696. https://doi.org/10.1038/s41467-023-43586-4
High throughput/arrayed microfluidic chips	Parallelized invasion or extravasation assays, drug screening on multiple lines/conditions	Medium (standardized inserts/arrays + automation)	High	End-point viability, automated imaging readouts, multiplexed secreted factor assays	*Sci Adv.* 2024 Aug 16;10(33):eadk0015. https://doi.org/10.1126/sciadv.adk0015
Sensors/omics integration (on-chip TEER, O₂, pH + downstream scRNA/proteomics)	Mechanistic mapping of steps (e.g., signaling during extravasation), real-time microenvironment readouts	Variable (depends on sensors & sample prep for omics)	Low–Medium	Continuous TEER/O₂/pH traces, live imaging, scRNA-seq, proteomics, metabolomics from captured cells	*Mater Today Bio.* 2025 Jun 2;33:101,925. https://doi.org/10.1016/j.mtbio.2025.101925
Computational/AI integration (in-silico + OOC data)	Predicts dissemination trajectories, optimizes device geometry, interprets high-dimensional readouts	Low physical complexity (computational) but high data/analysis complexity	High (scaling analysis)	Agent-based/CFD simulations, ML models trained on imaging/omics, predictive biomarkers	*J Vis Exp.* 2020 Aug 16;(162):10.3791/61654. https://doi.org/10.3791/61654

A relevant question to be addressed is which TOC platforms are promising tools for tailoring tumour therapies, and which have little chance of future use.

Among the TOC models that integrate PDTOs, several stand out for translational potential, but one class is considered especially promising for precision oncology. TOC models combining PDTO with vascular and immune microenvironments under dynamic perfusion, are highly promising because they faithfully recapitulate the tumour’s real-world complexity and have already shown early predictive correlation to patient outcomes [84, 85]. These platforms are more likely to reach clinical use first, because of *(a) Biological fidelity*. They reproduce drug diffusion, immune infiltration, and stromal signaling more realistically than any static model; *(b) Predictive power*. Early studies show alignment between chip-based responses and *actual patient outcomes; (c) Compatibility with biopsy-scale tissue*. They can operate with small input samples (needle biopsies), essential for real-world oncology; *(d) Quantifiable, high-throughput readouts*. Chips can include multiplex imaging, single-cell sequencing, or real-time impedance assays for rapid decision support; *(e) Scalability and automation potential*. Microfluidic arrays can be parallelized for multiple patients or drug combinations [86].

Application of bioprinting technologies [87], which allows for custom geometries, to TOC and OOC devices will provide relevant biomimetic disease models for high-throughput drug screening [88].

## The power of integrating tumor-on-chip and bioprinting technologies

TOC and bioprinted tumor are both advanced 3D in vitro cancer models, but they differ in fabrication method, structure, control, and purpose.

TOC is a microfluidic device that mimics the TME using channels, membranes, and controlled fluid flow, and allows for the precise regulation of oxygen, nutrients, interstitial flow, and mechanical stress. It can model tumor–stroma–immune interfaces and contain patient-derived cells or PDOs, arranged in microscale (millimetres) dimension [34, 35, 38]. It is ideal for real-time drug testing and excellent for studying metastasis, invasion, and vascular interactions.

A bioprinted tumor is made by layer-by-layer bioprinting of tumour cells and matrix materials that allow patient-specific tumor tissue reconstruction, in a meso–macroscale (hundreds of microns to centimetres) dimension. It is suitable for high-throughput drug screening and optimal for exploring 3D growth and ECM interactions, but the microenvironment control is limited and depends on bioink properties and diffusion, and less dynamic flow than in the TOC device [26, 41]. These two technologies are increasingly combined because, *(a) The tumour can be bioprinted in a form compatible with microfluidic chips*. Bioprinted tumour spheroids or tissue constructs can be fabricated in sizes that fit the microchambers of an OOC. *(b) OOCs support perfusion*. Perfusion provides nutrients and drug delivery similar to in vivo conditions, complementing bioprinted tumor models. *(c) Co-culture is feasible*. On a chip, the bioprinted tumour can interact with endothelial-lined channels, immune cells, stromal components, organ-specific tissues (lung, liver, gut, breast, etc.).

3D-printed TOC enables metastasis investigation through (a) multi-organ models containing miniaturized organs interconnected with the primary tumor to study how cancer cells migrate to distant sites; (b) incorporating microvascular networks [54] to study how tumors promote angiogenesis, intravasate to enter the circulation and extravasate at target sites; (c) high-resolution disease modeling characterized by predesigned arrangement of different cell types within.

The modular nature of 3D bioprinting and TOC platforms allows for customization of metastasis models and scalability of the experiments. Layer-by-layer bioprinting enables controlled placement of tumor cells, stromal cells, endothelial channels, and ECM gradients, permitting construction of metastatic niches (e.g., pre-metastatic niche, intravasation sites) [89]. Microfluidic TOC devices add physiologic flow, shear stress and soluble factor gradients that drive metastatic behaviors (migration, intravasation/extravasation) [35]. Combining standardized bioprinting protocols with microfluidic chip arrays enables dynamic simulation of tumor growth, metastatic dissemination, inter-organ interactions, and drug perfusion under physiologically relevant flow conditions [16].

Examples of introducing 3D-bioprinted tumour constructs into microfluidic/OOC platforms, are reported by,

- *Cao et al.,* who bioprinted perfusable hollow blood and (blind-ended) lymphatic vessel structures inside a cancer-mimetic hydrogel that contained tumour cells, and assembled that construct into a microfluidic TOC. Scientists tuned bioink composition to control vessel permeability, perfused the blood channel, and compared molecular/drug transport and tumour exposure across different vessel configurations. Combinations of blood/lymphatic vessel pairs produced different diffusion/clearance profiles for biomolecules and anticancer drugs, demonstrating how bioprinted micro-vasculature integrated into OOC enables more realistic transport studies [90].
- *Xie et al.*, who developed a 3D tumour array chip by incorporating electrohydrodynamic jet (E-jet) bioprinting of gelatin methacrylate (GelMA)-based cell-laden droplets, into a microfluidic format for functional testing. GelMA bioinks and E-jet printing were used to make reproducible 3D tumour spots compatible with chip-style handling. The platform was designed for drug screening under controlled delivery/perfusion conditions, showing feasibility of combining bioprinting and microfluidic testing [91].
- *Lu Z et al.* used a decellularized osteosarcoma (OS) ECM loaded with extracellular vesicles (EVs) from human bone marrow-derived stem cells and OS cells as a bioink for 3D printing of a micro-OS. This construct was integrated into a microfluidic device (with a recirculating perfusion system, which recreates bone marrow niche-like conditions, cell–cell and cell–matrix interactions, and fluid flow) to develop an OS-on-chip. The chip included bone marrow niches, cell‒cell and cell–matrix crosstalk, and circulation, thus providing a valuable research platform for studying OS biology, compared with traditional xenograft models, and enabling rapid treatment evaluation [92].
- *Cao et al. *described the fabrication of an improved tumour model consisting of bioprinted blood vessel and lymphatic vessel pair, embedded in a microfluidic bioreactor controlled by a micro-flow system, to achieve a dynamic microenvironment for cultured (MCF-7) BC cells in a 3D hydrogel matrix based on gelatin methacryloyl. The perfusable hollow blood vessel and the one end-blinded hollow lymph vessel were bioprinted separately with individually tunable permeability profiles matching those of their native counterparts. Researchers demonstrated that systems with different combinations of these bioprinted blood/lymphatic vessels exhibited varying levels of diffusion profiles for biomolecules and anti-cancer drugs. This unique in vitro tumor model may simulate the complex transport mechanisms of pharmaceutical compounds inside the TME [90].

Although microfluidic platforms and bioprinted models capture key tumor–organ interactions, currently they cannot fully replicate whole-body systemic physiology, including hormonal regulation, metabolism, and organ-organ crosstalk. Tumor evolution under systemic stress or long-term therapy is more complex than in static or short-term in vitro experiments. While in vitro models provide highly controlled, mechanistic insights, the multiscale complexity of human physiology and tumor evolution remains a key limitation for direct clinical translation.

As discussed below, the upcoming integration of tissue engineering platforms with OMICS data, molecular pathology, and ML algorithms, by providing mechanistic insight into tumor progression may enhance predictive power and accelerate drug development.

## The biological realism of printed models of primary tumor and organs sites of metastasis

Cell culture and animal models often fail to replicate the intricate architecture and microenvironment of human tumors. By using specialized printers that handle delicate biological materials, and digital designs for accurate reproduction of tissues and organs, bioprinting has made its way (sec. “Box 1”) offering unparalleled biological realism and potential for personalized medicine. Closely mimicking the native TME [93], 3D and, more recently, 4D bioprinted tumors provide powerful tools for understanding metastasis and developing precision oncology.

3D bioprinting of tumors involves the precise spatial deposition of bioinks containing living cells, ECM components, and growth factors to recapitulate the architecture and microenvironment [94, 95] of tumors and surrounding tissues, or of complex organs like lungs, liver, bone marrow or brains, the most common sites of metastasis (Fig. 1).

*Campbell and co-workers* provided compelling evidence that, by merging the fields of engineering and biological, bioprinting technology has a great translational impact when compared to non-bioprinted manually seeded (MS) cell cultures. To assess at the molecular level, the effects elicited by thermal inkjet bioprinting (TIB) in cancer cells, they performed viability, apoptosis, phosphorylation, and RNA sequence (RNA-seq) analysis of bioprinted MCF7 BC cells at separate timepoints post-bioprinting. Thermal inkjet bioprinting of MCF-7 BC cells induces profound molecular changes: viability after printing remains 76–77%, but apoptosis sharply increases (31% at 2 h, 64% at 24 h). RNA-seq analysis revealed ~ 9.7% of ~ 12,200 genes were significantly altered, including unique upregulation of LUCAT1, IL6, CCL26, and NRN1L. Phospho-MAPK profiling identified 21 hyperphosphorylated kinases in bioprinted cells (versus 9 in controls), pointing to activation of oncogenic signalling pathways (e.g., MAPK, p38, JNK, RSK, p53) related to drug resistance, survival, and proliferation. These results suggest thermal bioprinting may generate a cancer cell phenotype more stress-resistant and clinically relevant for drug-discovery models [96].

In a subsequent study, the same authors evaluated a novel combinatorial in vitro approach using TIB of human BC cells alongside conventional chemotherapy (palbociclib plus letrozole) and radiation treatment. They compared TIB cells *versus* manually seeded cells from BC lines (MCF-7, MDA-MB-231, MCF-10A) and measured viability after drug alone, radiation alone, and combined treatment. They found that the bioprinted cells exhibited higher resistance (i.e., higher viability) under the combined chemotherapy treatment compared with the manually seeded cells. Bioprinted cell configurations may better mimic in vivo tumour cell responses, thus providing a more realistic platform for evaluating anticancer combination therapies. The authors suggest that TIB models could improve preclinical drug/radiation synergy testing [97].

An example of 3D bioprinted tumour model was provided by *Maggiotto and co-workers*. They developed a 3D-bioprinted, perfusable, vascularized tumor model using (SK-N-AS) neuroblastoma cells co-cultured with MSCs and endothelial cells (HUVECs). Gelatin methacrylate (GelMA) bioink and sacrificial (Pluronic F-127) ink were used to fabricate thick constructs with hollow vascular channels. A custom perfusion bioreactor supported long-term culture (up to 3 weeks). This model allowed investigation of tumour–vessel interactions, including early metastatic processes [98].

4D bioprinting extends 3D bioprinting capabilities by incorporating “time” as a dynamic element. This technology uses stimuli-responsive materials, shaped by environmental cues (changes in pH, temperature, magnetic field, hypoxia, enzymatic cleavage, light wavelength), undergoing temporal transformations between multiple states, which enable modeling of the evolving nature of tumors, such as ECM remodeling, and angiogenesis simulating tumor responses to hypoxia, mechanical stress, and biochemical signals. Smart materials, used for 4D bioprinting, can sense and respond to environmental stimuli, that make time-based transformations possible [99, 100]. Therefore, 4D bioprinting adds to 3D printing, the dimension of time-dependent transformation, by exploiting the stimuli-responsive or self-modifying behavior embedded in the bioink or construct. Incorporating real-time changes, 4D bioprinting better replicates in vivo conditions providing insights into drug resistance and relapse mechanisms [99, 100]. Key features and applicability of 3D and 4D bioprinting in oncology research and clinical practice are highlighted in Table 5.

**Table 5:** Comparison between 3 and 4D tumor models

Aspect	3D tumor models	4D tumor models
Definition	Static structures that mimic the three-dimensional architecture of tumors	Dynamic models that incorporate time-dependent changes to replicate tumor evolution
Time dimension	Absent; models remain fixed after fabrication	Present; models can change over time in response to stimuli or internal mechanisms
Technological basis	Based on 3D bioprinting, spheroid cultures, or biocompatible scaffolds and hydrogels, often lacking responsive properties	Utilizes 4D bioprinting, smart materials, and stimuli-responsive systems, and shape-memory polymers that evolve under specific conditions
Complexity	Limited to architecture and cellular composition at the time of creation	Captures dynamic behaviors such as growth, invasion, metastasis, and treatment adaptation
Biological fidelity	Captures the spatial complexity but not the time-dependent dynamics	More accurately replicates the progression and adaptive nature of tumors
Applications	Understanding tumor structure. Basic drug screening	Studying tumor progression, metastasis, and drug resistance Advanced drug testing and therapy optimization
Application in drug testing	Provides a one-time snapshot of drug effects Useful for static drug screening and cytotoxicity studies	Enables real-time monitoring of drug efficacy, resistance mechanisms, and treatment timing
Cellular interaction	Static representation of interactions between cancer and ECM, stromal cells, vasculature	Simulates dynamic interactions, such as changes in immune response, angiogenesis, and ECM remodeling
Microenvironment	Recreates aspects like hypoxia and nutrient gradients but lacks adaptability	Models adaptive microenvironments that mimic real-time tumor plasticity
Immune response studies	Limited to static immune cell-tumor interactions	Enables dynamic studies of immune system-tumor interactions over time
Cost and scalability	Generally, more affordable, and scalable. It is expected to achieve broader scalability in clinical and industrial settings due to its relative maturity	Higher costs and complexity, requiring specialized equipment and expertise, and breakthroughs in materials science and engineering for large-scale adoption
Predictive accuracy	Moderate, better than 2D models but less representative of real tumor dynamics	High, closely mimics tumor evolution, improving translational success rates
Customization	Limited to initial printing parameters	Highly customizable, allowing patient-specific modeling and time-sensitive therapies
Challenges	Simpler to create but may lack relevance to dynamic in vivo conditions	More complex and resource-intensive to design and validate

While “5D bioprinting” is not yet widely addressed in the industry, several companies like Poietis, REGENHU, ROKIT Healthcare, Stratasys and CELLINK are at the forefront of 3D and 4D bioprinting technologies, enabling reconstructing tissues, organs, tumors and testing of treatments with a fair biological coherence.

Evolution of tumor bioprinting towards the fifth dimension represents a paradigm shift, allowing dynamic and highly realistic models of metastasis, incorporating data-driven customization. This property allows to create intelligent, adaptive, patient-specific biological systems informed by rich datasets and supported by AI and ML optimization.

5D bioprinting adds, to the 3D printing spatial framework, two rotational degrees of freedom of the print head or build platform, enabling curved, nonplanar deposition, anisotropic fiber alignment, graded material orientation, printing on free-form surfaces. Compared to 4D, 5D bioprinting involves enhancing mechanical design based on robotics and kinematics, where multi-axis motion improves the deposition trajectory and reduces interlayer defects of the construct and embedding additional layers of spatial and temporal complexity [101–103] (sec. “Box 2”). Its applications may advance the study of cancer progression and the development of personalized therapies, as described in the Table 6. Currently some research projects are explicitly focused on developing 5D bioprinting, which could pave the way for future commercial devices. An R&D grant/project is ongoing from 2024–25, called RoboPrint, involving NTN Robotics and Maxon companies, which aims to develop a robotic-arm-assisted 5D bioprinter. Although promising, especially to produce complex bone/organ scaffolds, the project is still in the research/development phase.

**Table 6:** Applications of 5D bioprinting in metastasis research and clinical practice

Category	Applications	Details
Metastasis research		
Tumor microenvironment	Creation of tumor microenvironment models	Enables studying tumor-stroma interactions and metastatic mechanisms
Drug screening	High-throughput drug screening platforms	Testing multiple anti-metastatic drugs in patient-specific conditions
Mechanistic studies	Investigation of metastasis pathways Insights into mechanobiology and immuno-metastasis interactions	Studying the roles of EMT, angiogenesis, and cell migration in a 5D dynamic model
Precision models	Printing patient-specific cancer tissues	Personalized insights into tumor behavior and metastasis progression
Clinical practice		
Personalized therapy	Fabrication of individualized tumor models Monitoring therapy resistance	Optimizing therapy strategies for metastatic cancers based on patient-specific bioprinted tissues
Surgical planning	Patient-specific surgical guides	Bioprinting metastasis-invaded organ models for precise surgical interventions
Therapeutic development	Testing and developing anti-metastatic therapies	Creating functional, vascularized tissues for validating novel therapeutic interventions in metastasis
Regenerative medicine	Integration with regenerative techniques to repair metastasis-damaged tissues	Bioprinting to replace or repair tissues affected by metastasis or cancer treatments
Bioprinted implants	Engineered implants for metastasis monitoring and localized therapy delivery	Implants capable of sustained drug release and real-time monitoring of metastatic progression

Integration of TOC and bioprinting [104] with AI and ML promises a breakthrough in cancer research and drug discovery [105]. AI and ML can improve data analysis and the utility of TOC and tumor bioprinting allowing for*, (a) data-driven insights:* ML models can analyze large datasets from TOC and bioprinted tumor-based experiments, such as cellular behavior, drug efficacy, and gene expression profiles [106]. AI algorithms can identify biomarkers and predict tumor behavior under different conditions; *(b) optimization of experimental design:* AI can optimize bioprinting parameters (bioink composition, cell density) and TOC designs for better physiological relevance. ML can predict experimental outcomes [107], reducing the need for trial-and-error approaches; *(c) drug discovery and screening:* AI can suggest compounds likely to be effective against specific tumor types based on data provided by TOC and bioprinted tumors. ML algorithms can identify synergistic drug combinations that might not be evident through traditional methods [107].

Future healthcare will take advantage of patient-derived tumor models enabling, *(a) personalized drug development:* AI-enhanced TOC and bioprinting can predict drug efficacy and toxicity based on clinical-pathological and molecular data, and provide real-time insight to refine therapeutic strategies, reducing costs and accelerating timelines [64]; *(b) clinical decision:* integration of AI with patient-derived TOC and bioprinted models can provide actionable insights for oncologists [108].

Prospectively, by integrating real-time data and AI-assisted biomimetic platforms, digital twins (i.e. a personalized virtual version of a patient used for real-time monitoring, prediction, and treatment optimization) would allow oncologists and researchers to test and optimize therapies safely and precisely before applying them to the actual patient.

## Molecular pathology and omics as cornerstones of human-relevant tumor-on-chip and bioprinted models

Molecular pathology and OMICS technologies play a pivotal role in enhancing the physiological relevance of TOC and bioprinting platforms for cancer research. These analytical tools allow comprehensive characterization of tumours at genomic, transcriptomic, proteomic, and metabolomic levels, capturing the molecular complexity and heterogeneity observed in human cancers [109, 110]. By integrating OMICS data, bioprinted tumour constructs and microfluidic TOC systems can be designed to accurately replicate patient-specific molecular signatures, TME interactions, and dynamic cellular responses. Molecular pathology further validates these models by comparing the histopathological, genetic, and molecular features of the models to those of real patient samples [111, 112]. This integrated approach ensures that the in vitro tumour models are not only structurally and functionally biomimetic, but also clinically relevant, enabling more predictive studies of tumor progression, therapeutic response, and resistance mechanisms.

The main question to answer is “*How can molecular pathology and OMICS technologies ensure that TOC and bioprinting systems are truly human-relevant platforms for modelling cancer biology and drug response?*” The paths to be implemented so that these investigative tools can be used to reproduce human cancer behaviour and to optimize the usefulness of the platforms involve:

1. **Validation of Biological Fidelity through Molecular Pathology**. Molecular pathology helps confirm whether the engineered tumour models, bioprinted tissues or TOC systems, reproduce the key molecular and histopathological features of human tumours.
2. **Histopathological comparison,** by using immunohistochemistry (IHC), in situ hybridization, and multiplex imaging (which combines IHC labelling many targets, high-resolution imaging, and computational image analysis to extract cell-by-cell and spatial information) to compare marker expression, cell morphology, and spatial organization, in the chip or printed tumour, with that of matching patient tissue [113]. Indeed, since multiplexed spatial profiling relies on inspecting thin (4–5 µm) specimens containing rare intact cells, which impairs cell phenotyping, recently *Yapp et al.*et al. developed a high-plex cyclic immunofluorescence method for 3D tissue imaging of sections eightfold to tenfold thicker, enabling accurate morphological assessment of several protein markers in intact tumour, immune and stromal cells [114].
3. **Tumour heterogeneity mapping.** Histopathology can identify whether the model reproduces intra-tumoral heterogeneity, such as, regions of necrosis, and cell phenotypes, which is essential for predicting therapy response [115–117].

By leveraging imaging and transcriptomics that can be applied on pathology slides *Lapuente-Santana et al.* proposed a computational framework to characterize the spatial biological contexture of tumours. A supervised ML model was trained on melanoma patients linking tile-level imaging features, extracted from haematoxylin and eosin (H&E) slides, to sample-level cell type quantifications, derived from RNA-seq data. The computational framework provides spatial cellular maps for any H&E image and converts them in spatial graphs to extract interpretable features capturing cellular architecture within the TME. Spatial features can distinguish four TME subtypes (desert, fibrotic, immune enriched, and immune enriched fibrotic) and can also be used to build an accurate prognostic model of 1 year survival status, identifying predictive positive and negative biomarkers [118].

- **OMICS for Deep Molecular Characterization**. OMICS platforms can provide comprehensive, quantitative molecular fingerprints of the TOC or bioprinted constructs, verifying that they mimic human tumors at multiple biological layers.
- **Genomics** ensures that the genetic background of tumor cells remains stable or deliberately reflects patient mutations (e.g., TP53, KRAS, EGFR). CRISPR and isogenic engineering let labs introduce or revert specific driver mutations (TP53, KRAS, EGFR) so the genetic background deliberately matches patient genotypes. CRISPR-engineered bioprinted tumours or PDO-on-chip can be sequenced to ensure genotype fidelity over time [119].
- **Transcriptomics (bulk or single-cell RNA-seq)** confirms that the gene expression profiles in the model match patient-derived tumors. Single-cell RNA-seq can reveal whether tumour-stromal-immune cell interactions are preserved, or if artificial culture conditions distort cell states [120].
- **Proteomics** measures signalling pathway activation, such as PI3K/AKT/mTOR and MAPK/ERK pathways, and abnormal protein–protein interactions that drive cancer progression [121, 122]. In addition, it is useful to identify post-translational modifications that might differ between in vitro and in vivo contexts [123].
- **Metabolomics** evaluates metabolic phenotypes (e.g., glycolysis, oxidative phosphorylation) under microfluidic flow or 3D bioprinted architectures. Furthermore, it can confirm that nutrient gradients and hypoxic zones mimic tumor metabolic stress in vivo [124].
- **Feedback Integration by which OMICS and Molecular Pathology Data can Drive Model Optimization.** Deviations from patient tumour profiles, assessed by integrating multi-omics and molecular pathology data, can guide refinement of bioprinting bioinks, microfluidic flow parameters, or co-culture conditions (e.g., by adding fibroblasts, immune cells, or endothelial layers). Importantly, multi-omics analysis, after drug treatment, helps determine if drug response pathways, such as those regulating apoptosis, DNA repair, and immune activation, are triggered consistently with clinical data and reflect clinical response or resistance mechanisms [125, 126].
- **Systems Biology Integration.** Combining OMICS and molecular pathology data through AI-driven integrative modelling (digital twins) enables, **(a) simulation of tumour dynamics under different drug regimens**, as summarized by *Peng et al.* which describes the advantages of using 3D culture models and organoid technology, for customized drug screening and drug resistance assessment [127]; **(b) real-time monitoring and prediction of tumour behaviour within the chip or bioprinted construct**, which can be achieved by combining high-frequency measurement of microenvironmental variables (O₂, pH, glucose/lactate, temperature), electrical/impedance signals (cell attachment, barrier integrity), and intermittent high-content imaging (morphology, fluorescent reporters). These complementary streams capture metabolic state, barrier function, invasion, proliferation, and short-term drug response [128]. *Matavosian et al.* reported recent advances in the field of real-time monitoring with a focus on automated assessment of time-sensitive bioink qualities such as mixing, pH, temperature, and viscosity by enabling the rapid optimization of printing parameters. Meanwhile, real-time monitoring of cell health, through concentration and viability, served as an indicator for bioactivity. Incorporating real-time monitoring into the bioprinting process, using closed-loop feedback, can improve the reproducibility and speed up transition of constructs into the clinic [129] **(c) mapping cancer biology in space, by using spatial OMICS and molecular pathology**, as reported by *Hsieh et al.*, which revised how spatial transcriptomics/proteomics/metabolomics are used to resolve TME heterogeneity and cell–cell interactions [130]. *Menon et al.* explored the use of microfluidic technologies for morpholomics and spatial omics, with a focus on single-cell and tissue characterization, and examined the use of microfluidics-assisted spatial barcoding with micrometer resolutions for the spatial profiling of tissue specimens [131]. Application of spatial transcriptomics in digestive system tumours was performed by *Huang et al.*, which showed how spatial transcriptomics can underpin the molecular validation of tumour architecture [132].

Taken together, these studies show the growing capacity to capture the molecular and spatial complexity of real tumours, which is exactly what model validation needs to reference.

5. **Ensuring Translational Relevance for Drug Testing**, by providing, **(a) Predictive validity**. Comparing drug responses in the model with known clinical outcomes validates that the model predicts patient responses accurately. **(b) Biomarker discovery**. OMICS profiling reveals mechanism-of-action markers and resistance signatures that can be used in clinical settings. **(c) Customization**. The use of patient-derived tumour cells and patient-specific omics data allows creation of precision TOC systems for individualized therapy screening [133, 134].

**Fig. 3:**
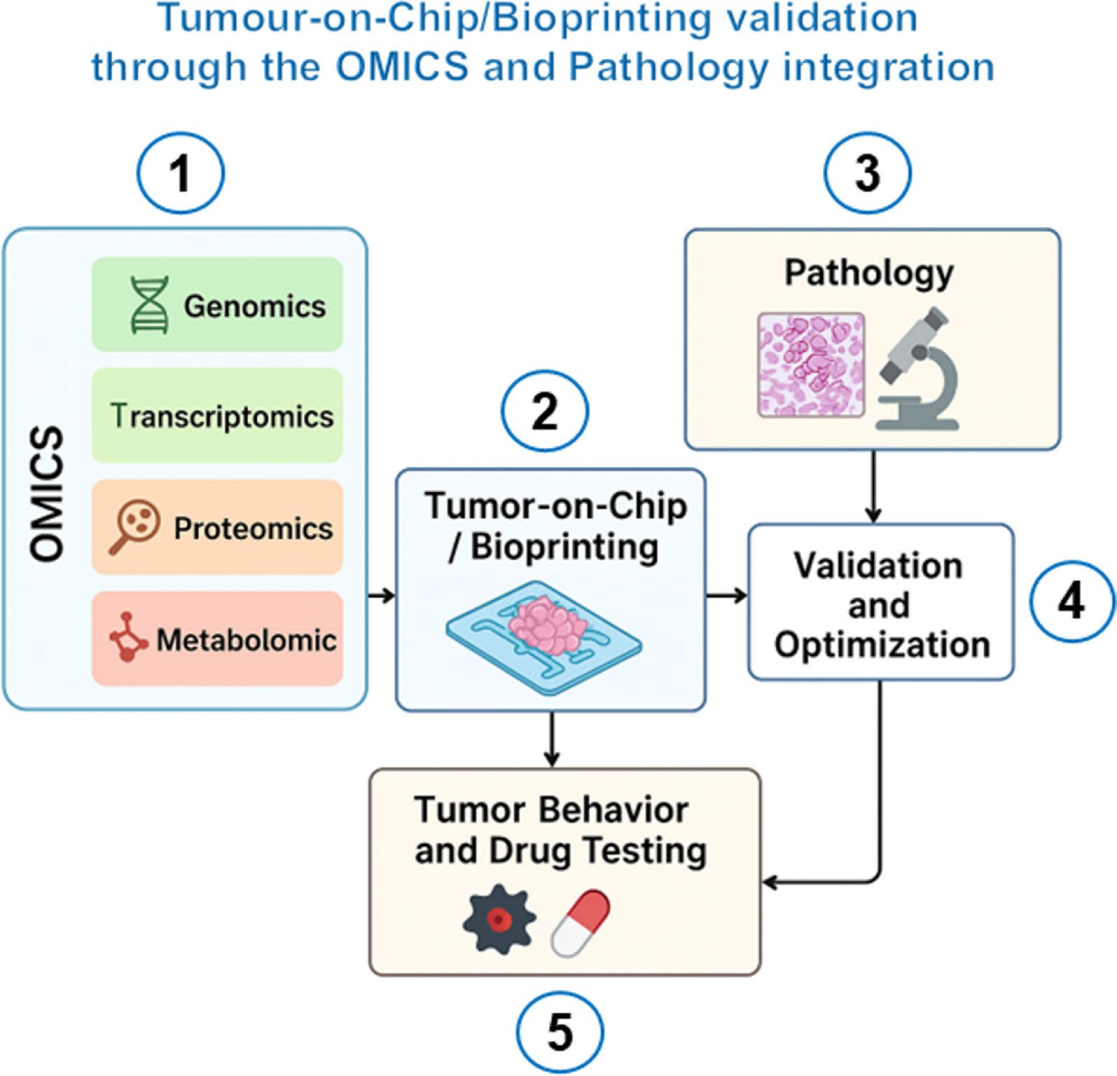
OMICS and Pathology Integration for Tumor-on-Chip/Bioprinting Validation. **1. OMICS (Left Panel–Light Blue Box)** represents the multi-layer molecular characterization feeding into TOC and bioprinting models. **Genomics** defines the genetic blueprint (mutations, copy number changes, structural variants) that determines tumour type and behaviour, and ensures that the model replicates key patient-specific genetic features. **Transcriptomics** captures gene expression patterns and cell-type signatures to evaluate how tumour and stromal cells respond to their microenvironment. It aims to verify that the engineered tumour expresses genes consistent with real tumour tissues. **Proteomics** measures protein abundance and signalling activity, validating functional pathway engagement (proliferation, apoptosis, angiogenesis). It aims to confirm biological processes are active as in vivo*.* **Metabolomics** profiles metabolic fluxes and pathway adaptations (e.g., glycolysis, hypoxia-driven metabolism). It aims to assess how nutrient gradients and oxygen levels in the chip replicate real tumor metabolism. These OMICS layers inform the input design and refinement of TOC/Bioprinted constructs. **2. Tumour-on-Chip/Bioprinting (Center Box–Light Blue)** represents the engineered 3D TME, integrating biological and microengineering technologies. TOC/Bioprinting platform combines tumour, stromal, immune, and endothelial cells in a dynamic 3D system equipped with a circulation-like flow, sustaining nutrient gradients, and provides a tool for tumour growth, drug testing, and microenvironmental interaction studies. **3. Pathology (Right Box–Beige)** represents molecular and histopathological validation of the model. Histology, immunostaining, and imaging are used to compare cell morphology, tissue organization, and biomarker expression with human tumours. Pathology confirms phenotypic realism at the tissue level. **4. Validation and Optimization phase (Middle Right Box–White)** represents the feedback loop that ensures biological accuracy and performance. It aims, (a) to integrate OMICS and pathology findings to refine bioink composition, cell ratios, microfluidic parameters, or environmental conditions, (b) to ensure ongoing model calibration to maintain genetic, molecular, and morphological fidelity. This step closes the loop between molecular data, observed tissue features, and system engineering. **5. Tumour Behaviour and Drug Testing (Bottom Box–Beige)** represents the application phase. Once validated, the model is used to assess tumour progression, invasion, metastasis potential, and treatment response. OMICS profiling after drug exposure identifies mechanistic responses and biomarkers of sensitivity or resistance. This phase aims to provide clinically relevant predictions for precision oncology and drug discovery

In summary, molecular pathology ensures phenotypic and histological fidelity, while OMICS ensures molecular and functional fidelity. Together, they can verify that TOC and bioprinted tumour models faithfully replicate patient-specific tumour biology, enabling reliable and translatable insights into tumour behaviour and drug efficacy. Figure 3 illustrates the steps to achieve Tumour-on-Chip/Bioprinting validation through the OMICS and pathology integration.

## Linking model systems and molecular read-outs to refine models and inform clinical decision making

Studies in which disease-specific (glioblastoma, lung, breast, gastric, osteosarcoma) engineered tumour models, either bioprinted or microfluidic/on-chip, were directly profiled using OMICS, or analysed via histology/molecular pathology (to validate/optimize the model), have provided compelling examples that directly link OMICS readouts to model refinement, allowing assessment of tumour transcriptional landscapes, and drug response prediction.

- A micro-engineered perfusable 3D-bioprinted glioblastoma (**GBM**) model was developed by *Neufeld et al.* to recapitulate the heterogeneous TME by using patient-derived GBM cells, astrocytes, and microglia, and perfusable blood vessels made of a bioink coated with brain pericytes and endothelial cells. Authors observed similar growth curves, drug response, and genetic signature of **GBM** cells grown in the 3D-bioink platform and in orthotopic cancer mouse models as opposed to 2D culture. This finding demonstrated that 3D-bioprinted model could be used to replace cell cultures and animal models, and to provide a platform for rapid target discovery, drug development and personalized therapy screening [135].
- *Lee et al.*, used a 3D angiogenesis-on-a-chip model that recapitulates human 3D angiogenic sprouting through coculture of ECs and fibroblasts, to investigate by scRNA-seq the heterogeneity of autophagy in endothelial cells (ECs) collected from the elongating sprouts on the chip. This study couples an on-chip angiogenesis model with scRNA-seq to map spatial functional heterogeneity, providing a direct example of on-chip-OMICS integration [136].
- By using hydrogel scaffold printed with an extrusion 3D bioprinter, *Zou et al.*, produced 3D-bioprinted **lung cancer** constructs containing a functional vasculature, and 2D cultures, to compare by RNA-seq conducted bioinformatics analysis, the transcriptional profiles of (A549) cancer cells derived from the two culture conditions. Results demonstrated transcriptional fidelity and the growth sustainability of the 3D-bioprinted tumour models relevant to assess drug response [137].
- *Tang et al.* leveraged bioprinting to create biomimetic, patient-derived tissues (PDT) that closely replicate the original tumour’s gene expression patterns and cellular compositions. RNA-seq, Whole-Exome Sequencing (WES), and flow cytometry were used for both primary tissues and PDTs to generate high fidelity PDTs via bioprinting. A multi-algorithm ML strategy was developed to produce reliable drug response (temozolomide, TMZ, and lomustine, i.e. chloroethyl-cyclohexyl-nitrosourea, CCNU) predictions based on gene expression data, which were validated using a **GBM** cell line, bioprinted PDTs, and bioprinted GBM-myeloid models containing either microglia or monocytes. Concordance of the recurrent clinical status to TMZ sensitivity, assessed through bioprinted PDTs, proved the biological fidelity of the model. Its predictive accuracy of drug responses was demonstrated by comparing the efficacy of TMZ and CCNU, in suppressing tumour cells, in PDTs and by identifying CCNU as the most effective chemotherapy for recurrent GBM patients. This study demonstrates the synergistic potential of integrating 3D bioprinted models, molecular (omics) profiling, which validates and informs model performance, and ML workflow for predicting and evaluating individual patient responses to treatment [138].
- *Lin et al.* developed a 3D bioprinted **OS** (3D-BPOS) model that contains OS cells and a shrouding ECM analogue. Photo-cross-linkable bioinks composed of gelatine methacrylamide and hyaluronic acid methacrylate mimicked tumour ECM. Multi-omics analysis, including transcriptomics and DNA methylomics, were used to determine differences between the 3D-BPOS and traditional models. Compared with 2D culture and tumour spheroids, the 3D-BPOS model showed significant changes in cell cycle, metabolism, adherens junctions, and other pathways associated with epigenetic regulation. The 3D-BPOS was more sensitive to therapies targeted to the autophagy pathway. Simulating the ECM yielded different osteosarcoma cell metabolic characteristics and drug sensitivity in the 3D-BPOS model compared to classical models, suggesting 3D printed osteosarcoma models can be used in basic and translational research [139].
- A printed **gastric cancer** (pGC) model was recently developed by *Choi et al.*, for preclinical chemotherapy evaluation, using extrusion-based 3D bioprinting technology and tissue-specific bioinks containing patient-derived tumor chunks. The pGC model retained the original tumor characteristics and enabled rapid drug assessment within 2 weeks of its isolation from the patient. The drug response-related gene profile of pGC tissues cocultured with human gastric fibroblasts was similar to the response of the patient’s tissues. The pGC model, overcomes the challenges associated with accurate drug evaluation in preclinical models, such as patient-derived xenografts, which lack the patient's stromal cells, and shows a remarkable similarity with patients in terms of response to chemotherapy and prognostic predictability [140].
- *Tung et al.* describe the development of a 3D bioprinted neurovascular unit (NVU) model, in a 96-well plate, containing patient-derived **GBM** cells, to study GBM tumour growth in a brain-like microenvironment, that is amenable for HTS. Extensive validation of the NVU-GBM model includes immunostaining for brain relevant cellular markers and ECM components, scRNAseq to detect physiologically relevant transcriptomics changes, and secretion of NVU- and GBM-relevant cytokines. The scRNAseq reveals changes in gene expression and cytokines secretion associated with wound healing/angiogenesis. The NVU-GBM model was used to test 18 chemotherapeutics in 4-point dose responses, and to evaluate anti-GBM efficacy. Results provide evidence of the pharmacological relevance of the model and robustness for HTS [141].
- *Shi et al.*, developed an optimized formulation of low-concentration collagen type I-based matrix bioink that was compatible with both **BC** cells and CAFs. They confirmed good printability of the bioink in a silk fibroin hydrogel-based support bath and then evaluated how matrix components affect BC cells and CAF phenotypes and oncogenic traits. This 3D bioprinted organotypic breast tumour models, that reconstructed several features of the TME, including angiogenesis, EMT, and invasion, demonstrates that the embedded bioprinting strategy can be applied to fabricate biomimetic models recapitulating tumour morphology and gene expression profiles [142].
- *Oliver *et al*.* used a 3D scaffold model obtained with bioprinted **GBM/mesenchymal stem cells** (**MSC)** cocultures, treated or not with conventional glioma therapies (chemotherapy, temozolomide, and/or radiotherapy), to analyze, at the transcriptomic level, the impact of human bone marrow-derived MSC (which acquire a CAF phenotype) on GBM growth and therapy response. GBM/MSC coculture and treatments impact on the GBM transcriptomic landscapes, in which mitochondrial DNA encoded genes were the top expressed genes, emphasizing the importance of mitochondria in the MSC/GBM interactions under 3D culture conditions. This is consistent with the central role mitochondria play in the interactions between cancer and the microenvironment. The coculture of MSC/CAF and GBM affects the expression of several genes, such as CXCL5 and COL16A, which are associated with invasiveness, tumorigenesis, and angiogenesis, and TREM-1, which is known amplify innate immune/inflammatory responses, and correlates with worse overall survival (OS) and progression-free survival (PFS) in GBM. TREM-1 was downregulated by temozolomide and/or radiation therapy, as assessed by the bioprinted model [143].
- Since in recurrent **GBM**, macrophages/microglia prominently contribute to the tumour mass, to investigate the impact of the microenvironment on the molecular program, *Tang et al.* developed a rapid 3D bioprinting method to compare the growth of **GBM stem cells** (GSCs) alone or with astrocytes and neural precursor cells in a hyaluronic acid-rich hydrogel, with or without macrophages. Bioprinted constructs integrating macrophages recapitulated patient-derived transcriptional profiles predictive of patient survival, maintenance of stemness, invasion, and drug resistance. Whole-genome CRISPR screening with tetra-culture bioprinted complex systems identified unique transcriptional profile and context-dependent pathways in GSCs, when compared to spheroid cell culture. Multicellular 3D bioprinted models provide a scalable and physiologic platform to interrogate drug sensitivity, cellular crosstalk, invasion, context-specific functional dependencies, as well as immunologic interactions [144].
- In the study by *Gebreyesus et al.* chips with different cell capacities were constructed to facilitate experiments with optimal profiling depth for different cell inputs. Specifically, an integrated proteomics chip (iProChip, 1–100 cells), and its extended version for single-cell capacity (SciProChip), were designed and coupled with data-independent acquisition (DIA) mass spectrometry (MS) as streamlined nanoproteomics (nanogram of cells) pipelines. This workflow illustrates the implementation of microfluidic devices with all-in-one functionality to achieve automated and streamlined proteomic preparation, which offers high sensitivity and reproducibility for limited input samples, enabling single-cell proteomics [145].

The reported research spans method development, such as on-chip sample preparation for proteomics, disease-specific models, such as those tailored to patients with GBM, GC, OS, lung or BCs, and examples that directly connect OMICS readouts to model refinement or drug-response prediction. These studies demonstrate the feasibility and potential benefits of integrating the described advanced platforms.

### Conclusions and perspectives

Improving the quality of care for the growing number of patients with advanced stage cancer has become challenging. Integrating TOC and OOC platforms, bioprinting technology, AI and ML assisted molecular biology, through an interdisciplinary framework, can support the fight against metastatic disease by enabling a biologically coherent setting for developing innovative and personalized solutions. While these tools are increasingly exploited for a comprehensive study of tumor biology and biomarker discovery, their use in clinical practice is far from reality, since costs and scalability remain a significant limitation, and unresolved issues need to be addressed (sec. “Box 3”).

The pairing of engineering, with OMICS and molecular pathology is one of the most promising routes towards true precision therapies for metastatic cancer. The reasons why combining these advanced research tools could be a force-multiplier involve:

- **Resolve cell states and trajectories**. Pairing chips/bioprinted tissues with scRNA-seq and single-nucleus (sn) RNA-seq can reveal which subpopulations (e.g., EMT-like, stem-like, immune-evasive clones) drive invasion or therapy resistance in the model, then those same signatures can be looked-for in-patient biopsies [146, 147].
- **Spatial context and molecular readouts**. Spatial transcriptomics/multiplexed imaging mapped onto 3D constructs or organoid chips allows to link gene expression to microanatomy (proximity to vessels, stromal niches), important for metastatic niche biology and for validating biomarkers discovered in bulk assays [148].
- **Proteomics and metabolomics for functional phenotype**. MS proteomics and metabolomics from chip effluents/tissue extracts supply orthogonal, actionable readouts (secreted factors, metabolic programs) that genomics alone misses, but which are crucial for drug target validation and PK/PD modelling [139].
- **Molecular pathology for clinical anchoring**. Integrating digital pathology (IHC/IF) and clinical molecular pathology ensures that the models recapitulate the histology and biomarker patterns seen in patient metastases, enabling more credible translational claims [149].

These integrated approaches have already shown impactful results, as demonstrated by *Klughammer and coworkers,* who developed a multi-modal map of metastatic tumors, by combining scRNA-seq, or snRNA-seq, of tumour biopsies from metastatic BC across diverse clinicopathological features, with a panel of four spatial expression assays (Slide-seq, MERFISH, ExSeq and CODEX) and H&E staining. This approach demonstrates the usefulness of integrating different experimental techniques to assess variability in cell type composition and expression, as well as spatial expression characteristics across clinicopathological and methodological diversity. Datasets like these create the ground truth for validating the biological fidelity of TOC and of bioprinted models [148]. Several 2024–2025 studies show proof-of-concept drug testing and mechanistic readouts [133].

#### Main technical and translational challenges to overcome for the practical exploitation of the alliance between the described cutting-edge technologies include:

- **Complexity *vs.* throughput trade-off**. The more physiologic the model (immune components, perfused vasculature, multi-organ links), the lower the throughput. This truth limits complicates drug screening and clinical timelines [150].
- **Recapitulating immune and stromal diversity.** Fully functional adaptive immunity (patient-matched T/B cell repertoires, the personalized adaptive immune landscape of an individual) and diverse stromal phenotypes are still hard to maintain long-term in vitro [151, 152].
- **Standardization and reproducibility.** Variability in bioinks, cell sources, device fabrication, and assay readouts makes cross-lab comparisons difficult. Benchmarks and reference datasets are needed [153].
- **Validation against clinical outcomes.** Ultimately models must predict patient responses (and failure modes) better than current preclinical tests, that requires retrospective and prospective clinical correlation studies [134].
- **Data integration and interpretation.** Multimodal OMICS combined with imaging, generates huge, heterogenous datasets that need robust integration (spatial mapping, batch correction, causal inference) and clinical annotation [154].

#### Accelerating the impact of the integrated approach on the clinical management of metastatic disease can be pursued by ensuring:

- **Production of bioprinters capable of large-scale manufacturing** with bioinks mimicking mechanical and biological properties of native tissues, and ensuring scalability meets the regulatory standards for safety, efficacy, and reproducibility.
- **Building standardized pilot pipelines** that combine PDOs/biopsies leading to TOC/bioprinted construct, which inform on treatment perturbation by combining scRNA-seq, spatial transcriptomics, targeted proteomics, and digital pathology.
- **Multi-site validation and biobanking.** Creation of linked biobanks containing patient’s organoids/primary cells with matched clinical data and multi-omics data is required, as well as performance of a blinded, multi-centre validation to verify whether model-derived biomarkers predict response or metastasis.
- **Data standards and open toolkits.** Agree on minimal metadata, Quality Control checks, and open analysis pipelines for multimodal data, so that ML/AI models trained on one dataset generalize.
- **Clinical decision support and trials.** Validated models integrated with omics pipelines could be used to stratify patients in adaptive clinical trials (e.g., test whether model-predicted combination therapy improves outcomes in metastatic cohorts). Regulatory commitments (FDA/EMA) are urgently needed for model validation [36, 155, 156]. Early interaction with regulators about model validation endpoints will smooth translation into clinical decision tools.
- **Personalized metastasis intercept strategies.** If models reliably predict the emergence of metastatic clones and effective combos, it will be feasible to treat patients prophylactically or with early targeted regimens tailored to the predicted escape pathways.
- **Patient consent and data linkage.** OMICS and patient clinical data require strong consent frameworks and secure data linkage for translational studies.
- **Cost affordability.** High-content multimodal pipelines are costly. Shared core facilities and consortia help democratize access.

In summary, combining TOC and 3D bioprinted tumor constructs with multimodal OMICS and rigorous molecular pathology creates a powerful pipeline for modelling metastasis mechanistically and for discovering clinically actionable biomarkers and drug combinations. The approach’s success hinges on standardization, patient-derived materials, integrated spatial/multi-omics readouts, and prospective clinical validation. In the next 3–5 years we should expect increasingly predictive preclinical platforms that can be tested in adaptive clinical trials, but getting there requires coordinated biobanking, benchmarking, and regulatory engagement together with a strong multidisciplinary network [149].

### Box 1. History of bioprinting technology and tumor bioprinting

Bioprinting technology began with the invention of 3D printing in the 1980s by Charles Hull [157]. Known as stereolithography, this technology involves curing photosensitive resin with UV light to build structures layer-by-layer. In the early 2000s, 3D printing was adapted for biological materials, introducing the concept of bio-printing, using bioinks [7]. Thomas Boland, one of the pioneers of bioprinting, modified an inkjet printer in 2003 to deposit cells and biomaterials [158]. 3D tumor bioprinting developed in the early 2000s and became a critical tool in reducing reliance on animal testing and in replicating TMEs [8]. Proof-of-concept studies demonstrated the feasibility of using bioinks laden with cancer cells to create 3D tumor-like structures [24, 25]. Advances in Bioinks and Materials dates back to the 2010s. Innovations in bioink chemistry allowed for better support, cell viability, and tissue functionality. Significant breakthroughs were achieved in replicating complex structures such as skin, cartilage, and vascular networks. Miniaturized, bioprinted organ models into OOC platforms were developed for drug testing and disease modeling [87, 88].

In the mid-2010s, the introduction of “time” as the fourth dimension led the transition to sophisticated 4D bioprinted tumors that incorporated multiple cell types, including stromal and immune cells, to replicate the TME. Specialized bioinks were developed to mimic the ECM and the biomechanical properties of tumors [94]. Tumor bioprinting has started to be used for high-throughput drug screening, enabling more accurate predictions of drug efficacy and toxicity [33, 34].

Bioprinted TOC models emerged in 2016, integrating tumor bioprinting with microfluidic systems to study tumor growth and drug delivery. By incorporating vascular networks into bioprinted tumors, nutrient delivery and oxygenation were ensured, further enhancing the realism of the models [159]. The manufacturing of functional organ prototypes dates to the 2020s showing promise for future transplantations. Bioprinting contributed to the regeneration of damaged tissues [101] and the development of patient-specific medical solutions, such as the production of patient-derived tumor models, to predict individual responses to therapies. Customized tumor models began incorporating immune cells to study cancer-immune interactions, speeding up immunotherapy research [95]. The advent of dynamic models of bioprinted tumors and metastatic organs enabled the simulation of tumor progression, response to treatments and development of drug resistance.

Importantly, bioprinters and bioprinting workflows have proven to produce tumour models that are explicitly suitable for HTS [160]. *Utama et al.* developed a bespoke drop-on-demand bioprinter (including inkjet and microvalve systems, which allow precise, controlled dispensing of very small liquid volumes) specifically designed for the high-throughput production of cancer spheroids, that prints reproducible spheroids in 96-well plates and demonstrated compatibility with automated handling and endpoint assays, explicitly designed for HTS [161]. The study proves that the bioprinting approach for embedded spheroid (containing SK-N-BE(2) neuroblastoma cells, or H460 non-small cell lung cancer cells, or U87vIII GBM cells) production enables HTP drug response analysis, and assessment of drug (doxorubicin) efficacy in inhibiting cell proliferation, through the determination of half maximal inhibitory concentration (IC50 values).

*Jung et al.* used a drop-on-demand/bespoke high-throughput phenotyping (HTP) platform (custom-built systems optimized for assay miniaturization, parallel sample processing, and integration with automated screening platforms) to produce large numbers of consistent 3D constructs for quantitative migration/invasion readouts, showing suitability for screening workflows [162]. By using (MCF7, MDA-MB-231) BC cells, and (H1299) non-small cell lung cancer cells, this study proved the feasibility of the 3D bioprinting HTP platform, coupled with tunable hydrogel systems, as a preclinical model for (i) real-time measurement of cell movement, (ii) molecular analysis of mechanisms underlying cell migration and invasion, and (iii) anti-metastatic drug testing.

*de Villiers et al.* described a pneumatic microextrusion approach to fabricate highly reproducible 3D melanoma (A375 cells) constructs for drug efficacy evaluations, with an eye toward analytical assays, such as viability assays (ATP, resazurin), automated imaging (high-content microscopy), migration/invasion quantification, multiplexed biomarker assays, and higher sample throughput for drug testing. This relatively simple, low-cost, and scalable method of producing and analysing 3D cell culture is useful when extrusion-printed matrices (hydrogels/ECM) are desired to build complex architecture (multi-material and multi-cell printing) with tunable mechanics (control over stiffness, porosity, and degradation) [163].

*González-Callejo et al.* used a bioprinted tumour–stroma coculture compatible with multi-well plate formats and HTS-style drug testing (multiplexed readouts). By combining a mix of breast decellularized ECM and methacrylated hyaluronic acid with BC-derived neoplastic and non-cancerous stromal cells. This platform provides a tool to replicate, in a tumor-stroma environment, the biological signalling pathways involved in tumour progression, and to assess therapeutic responses to chemotherapeutic agents [164].

Current trends and future directions in tumor bioprinting imply integration of AI and advanced imaging techniques enabling better design and analysis of tumor behavior and response to treatments [165] accelerating clinical trials.

### Box 2. 5D-bioprinting of tumors: advancing cancer research and treatment

5D bioprinting is a cutting-edge manufacturing that represents an advanced approach in tissue engineering [101, 102]. Based on the foundational printing principles of 3D printing (which consist of X, Y, Z = spatial axes along which material is deposited to build a structure layer by layer), the extra dimensions represented in 5D bioprinting are *(a) Dimension 4, i.e. curvature-aware orientation (multi-axis rotation).* The print head and/or build platform can rotate around additional axes. These rotations let material be deposited along curved paths rather than only stacking flat layers. Axis 4 consists in the rotation around one axis (e.g., tilting the print head or platform), which enables printing on curved surfaces, improving fibre orientation and reducing weak layer interfaces; *(b) Dimension 5, i.e. multi-axis freedom enabling orientation-controlled material properties*. A second rotational degree of freedom allows material to be deposited at precise 3D orientations at every point in the build. Axis 5 consists in the rotation around another axis, orthogonal to the 4th, which enables true anisotropic structuring, allowing fibres and filaments to follow natural stress lines, anatomical curves, or biological gradients. These peculiarities allow highly complex curved geometries that mimic natural tissue structures more accurately.

The 4D incorporates shape transformation over time, while 5D incorporates complex motion paths during printing, being focused on multi-axis mechanical precision. 4D bioprinting uses smart or stimuli-responsive bioinks (e.g., shape-memory polymers, hydrogels responsive to pH, temperature, light) [166, 167], while 5D uses composite bioinks optimized for structural and geometrical accuracy and mechanical strength. By integrating these dimensions, tumors can be replicated with greater accuracy, recreating their growth, evolution, and structural heterogeneity, and their interaction with surrounding tissues.

The main limitations to overcome for the practical applicability of the 5D bioprinting device involve the limited range of suitable bioinks and the difficulty in predicting their long-term transformations, the high cost of hardware and control systems, and the complexity of software management and path planning. Therefore, essential issues must be addressed for its widespread application in oncology research and practice, such as, *(a) costs and computational complexity to be lowered; (b) development of advanced bioink* to support diverse cell types and promote their interactions; *(c) production of tumor models suitable for HTS*; *(d) integration with advanced imaging and sensors; (e) transition from lab-scale bioprinting to clinical-grade applications, and development of standardized protocols for clinical use*. Future developments in biomaterials, ML, and imaging integration will further enhance the impact of 5D bioprinting in oncology research and practice.

### Box 3. Outstanding questions

- How to transfer the application of advanced technologies such as OOC and bioprinted tumors into clinical oncology practice? Could reducing the costs of acquiring and using AI-assisted TOC and bioprinting technologies help accelerate their introduction for clinical and therapeutic purposes?
- Might applying OOC and bioprinted tumors for pre-screening drug candidates, reduce failure rates in clinical trials and accelerate drug approval for clinical use?
- Might designing trials that incorporate patient-specific bioprinted tumors as companion diagnostics be sufficient to demonstrate their potential and transability into the clinical decision-making process?
- Might multi-center studies demonstrating improved outcomes in patients treated based on insights from these technologies speed-up their adoption for personalized treatment planning?
- Is it technically feasible and economically sustainable to incorporate OOC and bioprinted tumor models into the clinical decision-making process for each patient with advanced stage disease? Alternatively, could developing protocols to select patients that would benefit from the procedure favor their adoption into clinical practice?
- Could developing scalable manufacturing methods for reproducibility, and automating processes for drug screening or personalized medicine, accelerate their entering clinical guidelines?

## Data Availability

No datasets were generated or analysed during the current study.

## Abbreviations

AFP: Alpha-fetoprotein
AI: Artificial intelligence
ATAC-seq: Assay for transposase-accessible chromatin using sequencing
BC: Breast cancer
CAF: Cancer associated fibroblast
COL16A: Collagen alpha-1(XVI) chain
CTC: Circulating tumor cells
ctDNA: Circulating tumor DNA
CXCL5: C-X-C motif chemokine 5
DLS: Dynamic light scattering
ECM: Extracellular matrix
ELISA: Enzyme-linked immunosorbent assay
EMT: Epithelial mesenchymal transition
GC: Gastric cancer
GC–MS: Gas chromatography-mass spectrometry
GBM: Glioblastoma
IHC: Immunohistochemistry
LC–MS: Liquid chromatography-mass spectrometry
ML: Machine learning
MS: Mass spectrometry
MSC: Mesenchymal stem cells
ncRNA: Noncoding RNA
OOC: Organ on a chip
OS: Osteosarcoma
PDX: Patient-derived xenograft
PC: Prostate cancer
PD: Pharmacodynamics
PK: Pharmacokinetics
RANKL: Receptor activator of nuclear factor-κB ligand
RNA-seq: RNA sequencing
scDNA-seq: Single cell DNA sequencing
scRNA-seq: Single cell RNA sequencing
TME: Tumor microenvironment
TREM-1: Triggering Receptor Expressed on Myeloid Cells
TOC: Tumor on a chip
WB: Western blot
WGS: Whole genome sequencing

## Glossary

Bioinks: Specialized biocompatible and degradable materials used in bioprinting to create tissue-like structures. They consist of biomaterials (hydrogels, such as gelatin, alginate, collagen, fibrin, and hyaluronic acid); synthetic polymers (polyethylene glycol-PEG and polylactic acid-PLA); cells (stem cells, primary cells, cell lines); bioactive molecules (growth factors, peptides, or proteins) and cross-linkers to improve mechanical stability.
Bioprinting: Biofabrication technique that uses 3D printing technology to create living tissues or organ-like structures by precisely depositing cells, biomaterials, and bioactive molecules in a layer-by-layer manner, to mimic the structure and function of natural tissues.
Cas9: Enzyme that acts as molecular scissors, capable of cutting DNA at specific locations.
CCNU (Lomustine): 1-(2-chloroethyl)-3-cyclohexyl-1-nitrosourea). Alkylating agent (nitrosourea), which works interfering with DNA and RNA synthesis, leading to cancer cell death.
CRISPR: Clustered Regularly Interspaced Short Palindromic Repeats is a natural genetic system found in bacteria and archaea, that provides adaptive immunity against viruses, which has been adapted into a biotechnology tool for genome editing to precisely cut and modify DNA in living organisms.
Data-Independent Acquisition (DIA) Mass Spectrometry (MS): A proteomics data acquisition method in which the mass spectrometer systematically fragments all ions within predefined mass-to-charge (m/z) windows, rather than selecting only the most intense precursor ions (as in data-dependent acquisition, DDA), thereby enabling reproducible identification and quantification of both high- and low-abundance analytes.
E-jet bioprinting: Electrohydrodynamic jet bioprinting is an advanced 3D bioprinting technique that uses electrical forces to precisely deposit tiny droplets or continuous streams of bio-ink, allowing very high-resolution printing of cells, biomaterials, and tissue structures.
EVs: Extracellular Vesicles are small, membrane-bound particles secreted by cells into the extracellular environment.
Immune editing: The process by which the immune system interacts with developing tumors, shaping their immunogenicity through a dynamic balance of elimination, equilibrium, and escape phases. It describes how tumor cells are selected for survival based on their ability to evade immune detection, ultimately influencing tumor progression.
Isogenic engineering: Genetic engineering technique used to introduce, remove, or alter a specific gene or mutation within an otherwise genetically identical (isogenic) background, to allowing direct comparison of the effects of that change. Often done using tools like CRISPR-Cas9, Transcription Activator-Like Effector Nucleases (TALENs), or homologous recombination.
GelMA: Gelatin methacrylate is a gelatin-based bioink that provides mammalian cells with the essential properties of their native environment.
High-coverage whole-genome sequencing (WGS): Refers to sequencing the entire DNA of an organism, or tumor genome, at a depth sufficient to accurately detect nearly all classes of somatic variation: single-nucleotide variants (SNVs), small insertions/deletions (indels), copy number alterations (CNAs), structural variants (SVs).
Mass-spectrometry (MS) proteomics: A powerful analytical approach used to identify, quantify, and characterize proteins in complex biological samples. It combines protein chemistry, liquid chromatography, and mass spectrometry to study the proteome, i.e. the full set of proteins expressed by a cell, tissue, or organism.
ML: Machine learning is a subset of AI focused on developing algorithms and models that enable computers to learn from and make decisions or predictions based on data.
Mechanobiology: The study of how physical forces and mechanical properties of cells and tissues influence biological processes, such as development, physiology, and disease. It explores how cells “feel” and “respond” to mechanical cues, like stiffness, stretch, compression, or shear stress, and how these forces affect cell behavior, signaling, and gene expression.
Metastatic niche: Microenvironment that supports the survival, dormancy, growth, and proliferation of metastatic cancer cells after they have colonized the distant site. It forms after disseminated tumor cells have arrived at the secondary organ. Transforms the premetastatic “landing pad” into a functional “growth bed” that sustains metastatic outgrowth.
Multiplex imaging: Microscopy-based methods that detect many molecular markers such as proteins, RNAs, etc., simultaneously within a single tissue section, while preserving spatial context. Integrates multiple IHC, high-resolution imaging, and computational image analysis to extract cell-by-cell and spatial information.
OMICS: Term that encompasses various branches, genomics, transcriptomics, proteomics, metabolomics, epigenomics, lipidomics, glycomics and microbiomics (i.e. disciplines in biology whose names end in the suffix -omics) and uses advanced technologies and bioinformatics tools to generate and analyze large datasets.
OOC: Microengineered, biomimetic system that recreates the structure, function, and physiological environment of a tissue or an organ using living cells cultured within microfluidic devices. Microfluidics consists of channels that allow continuous flow of nutrients, oxygen, and drugs mimicking blood circulation. OOC typically includes multiple cell types organized in 3D.
Organoids: 3D miniatures derived from stem cells capable of self-organize and differentiate into various cell types replicating the tissue architectures and mimicking key structural and functional characteristics of real organs.
Patient-specific digital twins: Virtual models that replicate an individual patient’s biological systems, such as organs, tissues, or even cellular processes, based on their personal medical data (e.g., genetics, imaging, lab results).
Organotropism of metastasis: Non-random spreading and colonization of cancer cells to distant organs.
PDO: Patient-Derived Organoid is an organoid culture system established from normal, non-cancerous, tissue of a patient. Cell source is healthy tissue biopsy. PDO mimics the normal architecture and cell diversity of the corresponding organ.
PDTO: Patient-Derived Tumor Organoid is an organoid culture system generated from tumor tissue of a patient. Cell source is tumor biopsy, surgical specimen, or fluids like ascites pleural effusion and blood (CTCs). Retains cancer-specific genetic mutations, heterogeneity, and phenotypes.
Premetastatic niche: A microenvironment formed in a distant organ before tumor cells arrive. It is “prepared” by signals (such as exosomes, cytokines, and growth factors) secreted by the primary tumor, which condition the future site to support incoming cancer cells. Creates a favorable “landing pad” that makes it easier for disseminated tumor cells (DTCs) to survive and colonize.
Sacrificial bioprinting approach: A technique used in 3D bioprinting where a temporary (sacrificial) material is printed and later removed or dissolved to create hollow channels or spaces within a tissue construct.
snRNA-seq: Single-nucleus RNA sequencing is a specialized method for profiling gene expression at the level of individual nuclei, rather than whole cells. It’s particularly useful for tissues that are difficult to dissociate or where cells are fragile (like the brain).
TALEN: Transcription Activator-Like Effector Nuclease is a gene-editing technology consisting of transcription activator-like effectors (TALEs), proteins engineered to recognize specific DNA sequences, fused to a nuclease domain, typically derived from the FokI restriction enzyme.
Tumor architecture: Structural and spatial organization of the different components within a tumor, including how cancer cells, stromal cells, blood vessels, and ECM are arranged and interact.
Tumor microenvironment: Complex ecosystem of non-cancerous components surrounding and interacting with tumor cells, including stromal cells, immune cells, blood vessels, extracellular matrix, and signaling molecules.
TOC: Specialized form of OOC designed to recreate the TME, including cancer cells, stromal cells, immune cells, extracellular matrix, and fluid dynamics, within a microfluidic platform.
